# Synthesis of 3-Methoxy-6- [(2, 4, 6-trimethyl-phenylamino)-methyl]-phenol Schiff base characterized by spectral, in-silco and in-vitro studies

**DOI:** 10.1016/j.heliyon.2022.e10070

**Published:** 2022-08-05

**Authors:** Suganya Murugan, Jayasudha Nehru, David Stephen Arputharaj, Anaglit Catherine Paul, Prasanth Gunasekaran, Necmi Dege, Emine Berrin ÇINAR, Kasthuri Balasubramani, Jose Kavitha Savaridasson, Abdullah G. Al-Sehemi, Venkatachalam Rajakannan, Madhukar Hemamalini

**Affiliations:** a Department of Chemistry, Mother Teresa Women's University, Kodaikanal, India; b Department of Physics, PSG College of Arts and Science, Coimbatore, India; c Centre of Advanced Study in Crystallography and Biophysics, University of Madras, Chennai, India; d Department of Physics, Ondoku Mayis University, Samsun, Turkey; e Department of Chemistry, Government Arts College (Autonomous), Karur, India; f Research Centre for Advanced Materials Science, King Khalid University, Abha 61413, Saudi Arabia

**Keywords:** Crystal structure, DFT, Molecular docking, Swiss ADME studies, NCI index, Anti-bacterial activity and MTT-assay

## Abstract

The structure of the title compound (I) (C_17_H_19_NO_2_)_2_ the Schiff base, {3-Methoxy-6-[(2,4,6-trimethyl-phenylamino)-methyl]-phenol} was characterized by ^1^H, ^13^C NMR, UV–VIS and IR spectroscopic techniques. The crystal structure was determined by X-ray analysis. The compound (I) was crystallized in the Monoclinic space group P2_1_/c, with a = 25.9845 (12), b = 7.3318 (4), c = 16.3543 (8) Å, β = 100.713(°) (4), and Z = 8. The intermolecular interactions of the compound (I) was analyzed using Hirshfeld surface and Fingerprint analysis. Based on the crystal-void calculation, the volume of the void and surface area of the Schiff base compound (I) was described. The frontier molecular orbital energy gap reveals charge transfer interactions involving donors and acceptors. The invitro studies on antibacterial property of the title compound shows best MIC value for *Staphylococcus aureus* and the compound effect on MTT assay on A549 lung cancer cell line. The molecular docking result shows that the compound has good molecular-level interaction with anticancer drug target having good interactions with active site residues. The non-covalent interactions in the protein-ligand complex were well established from NCI analysis.

## 1 Introduction

Schiff bases are compounds with azomethine [-C=N-] groups in their structure and can be obtained from the condensation of primary amines with active carbonyl compounds. Since a variety of methods for the synthesis of imines have been described [1]. Imine and its derivatives have been shown to play an important role in living organisms [2, 3, 4], and biological applications [5, 6], co- ordination chemistry, catalysts, polymer stabilizers, corrosion inhibitors, dyes and pigments [7, 8, 9]. Schiff base metal complexes have greater biological activity compared to free organic compounds [[10], [11]]. Schiff bases are used in the photo stabilization of poly (vinyl chloride) polymers against photo degradation by ultraviolet radiation. Herein, we have discussed the synthesis, crystal structure, spectral characterization, in-vitro anticancer, antibacterial analysis, molecular docking, in-silico Swiss ADME studies of the 3-Methoxy-6-[(2, 4, 6-trimethyl-phenylamino)- methyl]-phenol Schiff base compound (I). The complete structural information of this Schiff base compound (I) has been gained from single X-ray diffraction and their intermolecular interaction were examined by Hirshfeld surface and NCI analysis. The chemical hardness, softness, electrophilic and nucleophilic region of the Schiff base was analyzed by Frontier Molecular Orbital (FMO) energies.

## 2 Experimental procedure

### 2.1 Materials & methods

2,4,6-timethylphenylamine, *O-*Vanillin and ethanol were purchased from Sigma Aldrich (commercially available) and used without further purification.

### 2.2 Crystallization and X-ray structure determination

**Scheme 1:**
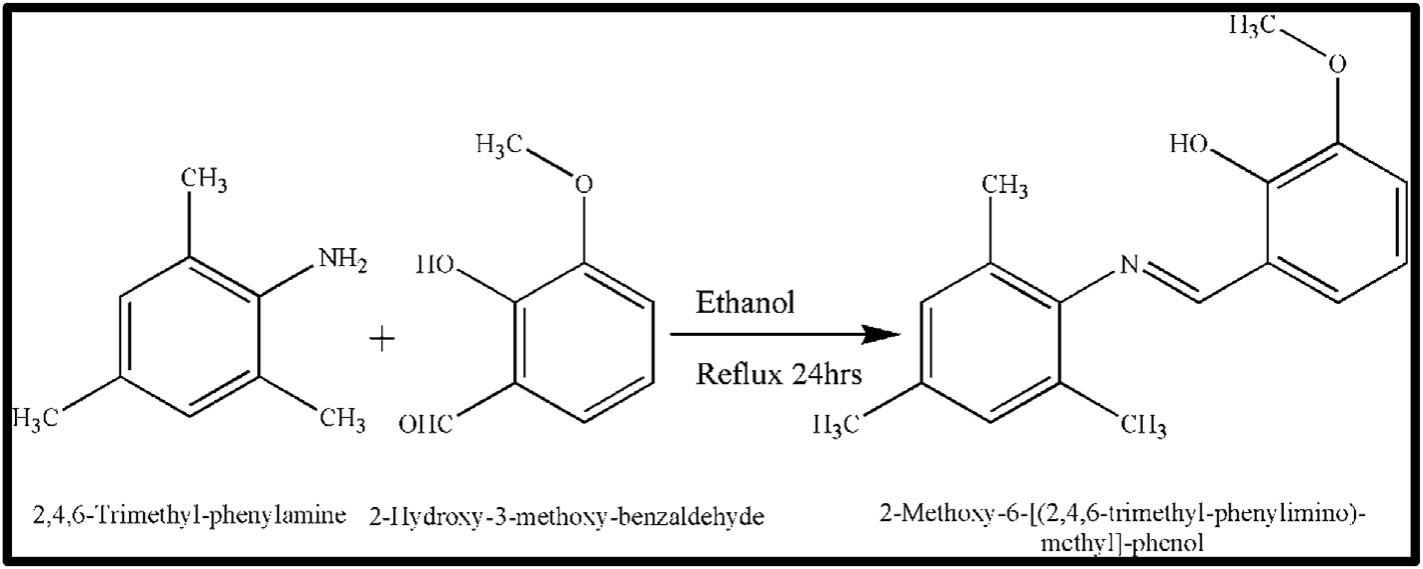
Synthesis of Schiff base compound (I).

A Schiff base ligand was synthesized by condensing 2,4,6-trimethylphenylamine (0.01 mol)and vanillin (0.01 mol) in ethanol, where the mixture was refluxed and stirred for 24 h, then kept for crystallization. After a few days yellow colour needle shaped crystals of Schiff base compound (I) was obtained as shown in Scheme 1.

Data collection of the title compound (I) was performed using STOE IPDS 2 diffractometer provided with graphite monochromatic with MoKα radiation at 296K. The structure solution was carried out using SHELXS–97 and the structural data were refined by full-matrix least-squares methods on F^2^ using the SHELXL–97 program package [12, 13, 14]. The carbon and oxygen bound hydrogen atoms were refined as riding model C-H = 0.93–0.96Å and O–H = 0.82Å.The crystal data and structure refinement parameters details were given see Table 1.

**Table 1:** The crystal data and structure refinement parameters.

Crystal data	
Chemical formula	C_17_H_19_NO_2_
*M*r	269.33
Crystal system, space group	Monoclinic, *P*2_1_/*c*
Temperature (K)	296
*a*, *b*, *c* (Å)	25.9845 (12), 7.3318 (4), 16.3543 (8)
*β* (°)	100.713 (4)
*V* (Å^3^)	3061.4 (3)
*Z*	8
Radiation type	Mo*Kα*
*μ* (mm^−1^)	0.08
Crystal size (mm)	0.59 × 0.34 × 0.09
**Data collection**	
Diffractometer	STOE *IPDS* 2
Absorption correction	Integration
	(*X-RED32*; Stoe&Cie, 2002)
*T*_min_, *T*_max_	0.974, 0.993
No. of measured, independent and	19262, 5464, 2882
observed [*I* > 2*σ*(*I*)] reflections	
*R*_int_	0.059
(sin *θ*/*λ*)_max_ (Å^−1^)	0.598
**Refinement**	
*R*[*F*^2^> 2*σ*(*F*^2^)], *wR*(*F*^2^), *S*	0.058, 0.136, 1.03
No. of reflections R,WR2,S	5464 0.0579, 0.1356, 1.03
No. of parameters	371
H-atom treatment	H-atom parameters constrained
Δ*ρ*_max_, Δ*ρ*_min_ (e Å^−3^)	0.12, −0.11
CCDC Number	2109553

The Crystallographic Data has been deposited in Cambridge Crystallographic Data Centre, with CCDC reference number 2109553. The data can be obtained available free of charge from http://www.ccdc.cam.ac.uk/conts/retrieving.html or from the Cambridge Crystallographic Data Centre (CCDC), 12 Union Road, Cambridge CB2 1EZ, UK; fax: +44 (0)1223336033; email: deposit@ccdc.cam.ac.uk.

### 2.3 Spectral studies

FTIR Perking Elmer Spectrophotometer using KBr pellet and the spectrum was recorded in the scan range of 4000-400 cm^−1^ region. UV-Visible absorption spectrum was recorded using SHIMADZU model UV-2600 Spectrophotometer in the wavelength range of 200–800 nm in ethanol solution with quartz cell of 1.0 cm path length to give resolution at 0.54 × 0.54 μm^2^ pixels [15]. Solution state ^1^H &^13^C Nuclear Magnetic Resonance spectra were recorded on Bruker Advance III HD Nanobay 400 MHz NMR spectrometer. Samples were analyzed in deuterated DMSO and the chemical shifts were relative to tetramethylsilane (TMS) as a reference [16].

### 2.4 Computational study

The quantum chemical calculations of the title compound (I) have been performed by DFT- B3LYP/6-311G basis set [17, 18], using the Gaussian 09W program [19]. Hirshfeld surface and their two-dimensional fingerprint plots were useful tools for describing the surface characteristics of the crystal structure using the Crystal Explorer 17.5 [20] package. The *d*_*norm*_values are mapped on to the Hirshfeld surface by using a red-blue-white colour, where red regions correspond to shorter contacts (-*d*_*norm*_value), the blue regions correspond to longer contacts (+*d*_*norm*_value), and the white regions correspond to the contacts around the van der Waals radii.

### 2.5 In-silico ADME predictions

The various physicochemical features and pharmacokinetic descriptors such as LogP, topological polar surface area (TPSA), number of hydrogen bond donors (HBD), acceptors (HBA), and number of rotatable bonds were calculated for Schiff base compound (I) through the online tool Swiss ADME [21, 22, 23] server.

### 2.6 *In-vitro* anti-bacterial studies

The antimicrobial activity was done to find the efficacy of the Schiff base compound (I) using well diffusion method and the concentration was fixed using Minimum inhibitory concentration (MIC) method. The following test microorganisms were obtained from Microbiology Laboratory, Department of Microbiology, Sacred Heart College (Autonomous), Tirupattur, Tamilnadu, India. Gram positive bacteria (*Actinobacteria, Bacillus Subtilis* and *Staphylococcus aureus*) *and Gram negative bacteria (Escherichia coli* and *Pseudomonas aeruginosa)* [24, 25]The cell Suspension of each microorganism was made by transferring aloop full of test organism from culture to 10 mL of sterile normal saline solution and make a standard stock. The compound was dissolved in dimethyl sulphoxide (DMSO) to prepare a stock Solution at 10 mg/mL and prepared as different concentrations viz 25, 50, 75 and 100ppm and labelled.

### 2.7 MTT assay

A549 cells were purchased from the National Centre for Cell Sciences (NCCS) Pune, maintained in Dulbecco's Modified Eagle Media (DMEM) with high glucose medium supplement with 10% FBS. Cells incubated to a humidified environment at 37 °C containing 5% CO2. The MTT assay was used to determine the *in vitro* cytotoxicity of MH-3 in A549 cells. A549 cells were seeded in 96-well plates (Costar, IL, U.S.A.) at a density of 2 × 10^4^ cells mL^−1^, respectively [26]. After incubating with complete DMEM (containing 10% FBS) for 24 h under standard conditions (37 °C, 5% CO_2_) in the dark, different concentrations of A549 (100 μL) were diluted in DMEM and added to the wells. The cells were cultured in a dark environment for another 24 h. After the cells adhered, the A549 solution diluted with DMEM solution was added. After that, A549 suspensions were replaced by 100 μL fresh DMEM and then washed with PBS buffer; 20 μL of freshly prepared MTT solution was added to each well. After incubation at 37 °C for 4 h, the supernatant was removed, and 100 μL of dimethyl sulfoxide (DMSO) was added. The plate was gently shaken for 10 min at room temperature to dissolve all the precipitates formed. ARobotnik Elisa Microplate Reader recorded the absorbance intensity at 490 nm. The cell proliferation was photographed using an Olympus CKX-53 microscope (Japan).

### 2.8 Molecular docking

Molecular docking studies were carried out through AUTODOCK TOOLS 4.2 software [27]. To evaluate the molecular level and structure based antibacterial, anticancer activity of the compound, drug targets from *E. coli*, Human dihydrofolate synthase (chemotherapy cancer drug target) were used in the docking study. The crystal structures of drug targets such as Human dihydrofolate reductase (DHFR) (PDB ID: 4M6K), *E. coli* beta-ketoacyl-acp synthase iii (PDB ID: 1HNJwere downloaded from protein data bank *according to the high resolution and with substrate or inhibitor complexed structure criteria)* [28, 29]. The protein structures were prepared for docking by removing additional water molecules, co-crystallized structures and chains leaving necessary molecules related to its activity. The protein PDBQT file containing added hydrogen, atom types and kollmann charges and ligand PDBQT file containing defined root atoms and number of torsions were prepared using AUTODOCK TOOLS GUI. The grid box parameters were set manually by positioning the grid box around residues having catalytic activity or substrate binding activity. The rigid receptor docking was performed for compound against target proteins using genetic algorithm as search parameter and Lamarckian GA as output generator. For each protein-ligand docking, ligand-receptor complex was chosen based on least binding energy, inhibition constant value, interactions with important residues. The complexes were downloaded and visualized using PyMOL and Poseview*, Ligplot* [30, 31, 32] interaction images were prepared.

## 3 Result and discussion

### 3.1 Crystal structure

**Figure 1:**
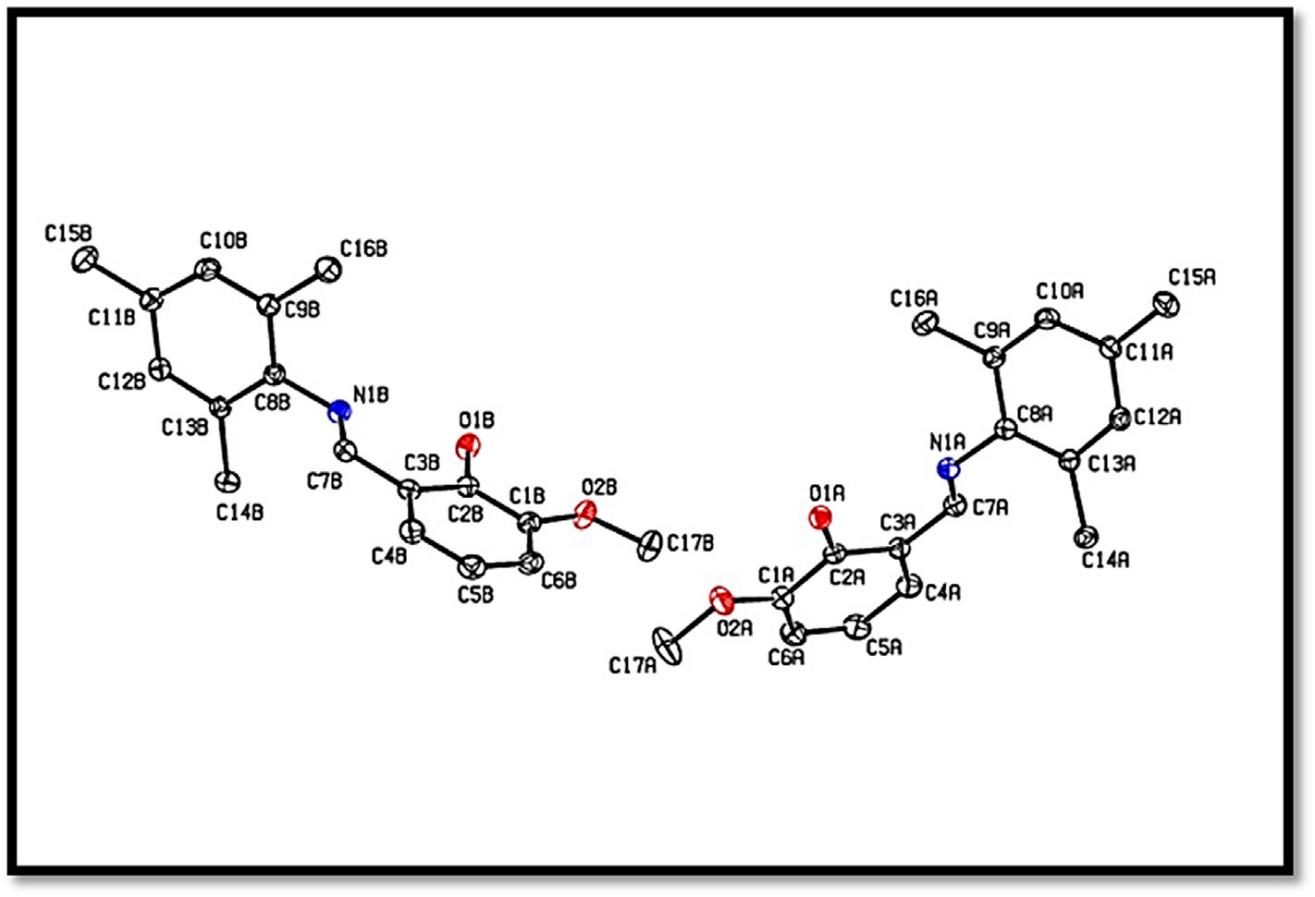
The asymmetric unit of the title compound I. Displacement ellipsoids are drawn at the 30% probability level.

**Figure 2:**
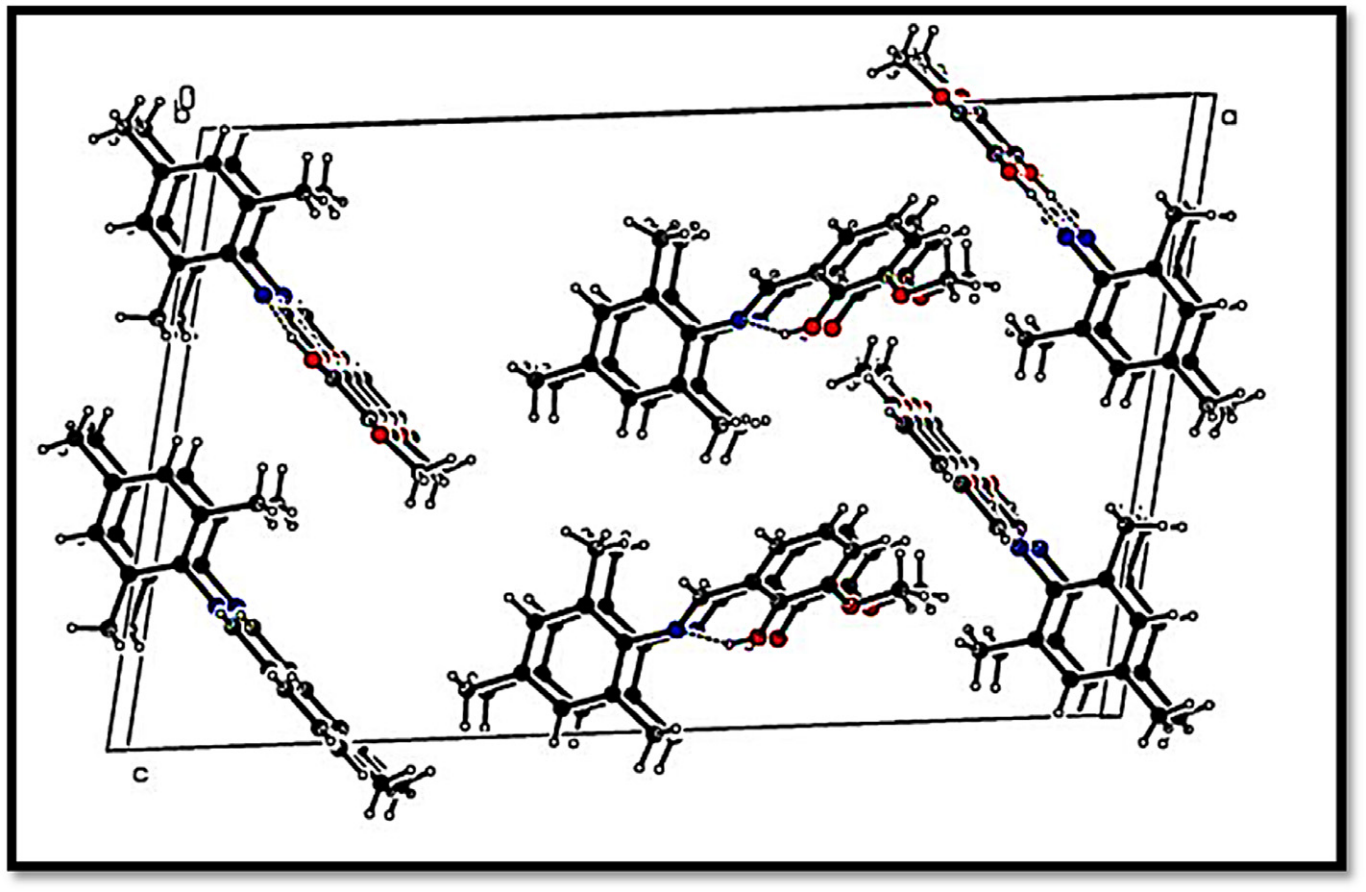
C---H…π interactions of the Compound (I).

The ORTEP ellipsoidal plot of Schiff base compound (I), C_17_H_19_NO_2_, crystallizes in the monoclinic space group P2_1_/c with two independent (A & B) molecules in the asymmetric unit (see Figure 1). The conformation of the C7A-N1A and C7B-N1B imine bond is *E*. The molecule is non-planar, with dihedral angle between the aryl rings (C1A---C6A) & (C8A—C13A)/(C1B–C6B) & (C8B–C13B) of 60.35 (13)^o^ Å and 86.27 (13)^o^ Å.The C7A-- N1A and C7B–N1B bond lengths are consistent with the presence of a double bond [1.278 (3) Å and 1.271 (3) Å], while the C2A—O1A, C1A— O2A, C2B–O1B & C1B–O2B bond lengths [ranging from 1.353(3) Å to 1.365 (13) Å] are consistent with a single bond (Table 2). The crystal packing of molecules was stabilized by O-H…N, C-H…O, C-H…N and C-H…C type of inter and intra molecular interactions. Interestingly, there were three bifurcated hydrogen bonding framed from C-H…O and C-H…C interactions and influenced more in the crystal packing stability. The observed bifurcated H-bonds were “Y” type and classified as three-center hydrogen bonds. The sum of the bond angles in the C12A-H12A...O1A/C2A, C6A -H6A...O1B/C2B and C6B-H6B...O1A/C2A interactions were 304.5^o^, 322.1^o^ and 330.4^o^ respectively and these values exhibit significant deviations from the planarity. Among the intra and inter molecular interactions, O-H…O, and C-H…O type of interactions were considered to be the strongest, and their geometrical parameters were listed (see Table 2 and see Figure 2).

**Table 2:** Geometrical parameter of the Title compound (I).

Bond Length (Å)			Bond Angle (deg)		
Parameters	Experimental	B3LYP/6-311G	Parameters	Experimental	B3LYP/6-311G
O1A-C2A O2B-C1B N1A-C7A N1B-C7B O2A-C1A C8A-C13A C13B-C12B C13B-C14B C2A-C1A C2B-C1B C13A-C14A C3A-C7A C12B-C11B C3B-C4B C9A-C10A C12A-C11A C11B-C15B C11A-C15A C9B-C10B C4A-C5A C6B-C5B O1B-C2B O2B-C17B N1A- C8A N1B- C8B O2A- C17A C8A - C9A C13B- C8B C2A- C3A C2B- C3B C13A- C12A C3A- C4A C8B- C9B C7B- C3B C1B- C6B C9A- C16A C11B- C10B C11A- C10A C1A- C6A C9B- C16B C4B- C5B C6A- C5A	1.353 (3) 1.365 (3) 1.278 (3) 1.271 (3) 1.360 (3) 1.394 (3) 1.377 (3) 1.506 (3) 1.400 (3) 1.398 (4) 1.512 (3) 1.443 (3) 1.384 (4) 1.394 (4) 1.380 (4) 1.381 (4) 1.508 (4) 1.511 (4) 1.376 (4) 1.362 (4) 1.385 (4) 1.354 (3) 1.424 (3) 1.427 (3) 1.433 (3) 1.414 (4) 1.396 (3) 1.393 (3) 1.391 (3) 1.389 (3) 1.391 (3) 1.404 (4) 1.388 (4) 1.451 (4) 1.375 (4) 1.510 (4) 1.383 (4) 1.376 (4) 1.367 (4) 1.513 (4) 1.355 (4) 1.381 (4)	1.367 1.380 1.299 1.299 1.380 1.410 1.402 1.514 1.414 1.414 1.510 1.450 1.397 1.415 1.402 1.402 1.512 1.512 1.396 1.383 1.407 1.367 1.460 1.427 1.427 1.460 1.410 1.410 1.414 1.414 1.396 1.415 1.413 1.450 1.391 1.514 1.402 1.397 1.391 1.510 1.383 1.407	C1B-O2B-C17B C7B-N1B-C8B C13A-C8A-C9A C9A-C8A-N1A C12B-C13B-C14B O1A-C2A-C3A C3A-C2A-C1A O1B-C2B-C1B C12A-C13A-C8A C8A-C13A-C14A C2A-C3A-C7A C9B-C8B-C13B C13B-C8B-N1B N1B-C7B-C3B C2B-C3B-C7B O2B-C1B-C6B C6B-C1B-C2B C10A-C9A-C16A C11A-C12A-C13A C10B-C11B-C12B C12B-C11B-C15B C10A-C11A-C15A O2A-C1A-C6A C6A-C1A-C2A C10B-C9B-C16B C11A-C10A-C9A C5B-C4B-C3B C9B-C10B-C11B C1A-C6A-C5A C7A-N1A-C8A C1A-O2A-C17A C13A-C8A-N1A C12B-C13B-C8B C8B-C13B-C14B O1A-C2A-C1A O1B-C2B-C3B C3B-C2B-C1B C12A-C13A-C14A C2A-C3A-C4A C4A-C3A-C7A C9B-C8B-N1B C13B-C12B-C11B C2B-C3B-C4B C4B-C3B-C7B O2B-C1B-C2B C10A-C9A-C8A C8A-C9A-C16A N1A-C7A-C3A C10B-C11B-C15B C10A-C11A-C12A C12A-C11A-C15A O2A-C1A-C2A C10B-C9B-C8B C8B-C9B-C16B C5A-C4A-C3A C1B-C6B-C5B C4B-C5B-C6B C4A-C5A-C6A	116.8 (2) 119.2 (2) 120.8 (2) 117.2 (2) 121.0 (2) 122.4 (2) 119.6 (3) 118.0 (2) 117.8 (2) 123.0 (2) 121.1 (2) 121.6 (2) 119.3 (2) 123.1 (3) 120.7 (2) 125.3 (3) 119.0 (3) 121.0 (3) 123.0 (3) 117.1 (3) 121.1 (3) 121.7 (3) 125.6 (3) 119.7 (3) 120.7 (3) 123.2 (3) 120.5 (3) 122.9 (3) 120.9 (3) 121.3 (2) 118.7 (3) 121.9 (2) 117.7 (2) 121.3 (2) 118.0 (3) 122.0 (2) 120.0 (2) 119.2 (2) 119.3 (2) 119.6 (3) 119.0 (2) 122.8 (2) 119.4 (2) 119.8 (3) 115.7 (2) 118.2 (2) 120.8 (2) 122.4 (2) 121.8 (3) 117.0 (2) 121.3 (3) 114.7 (3) 117.9 (2) 121.4 (3) 120.3 (3) 121.0 (3) 120.1 (3) 120.2 (3)	119.6 123.1 120.7 117.4 126.2 121.5 119.6 118.7 118.2 122.1 120.4 120.7 121.6 122.2 120.4 125.0 119.6 120.9 122.3 117.9 121.2 120.7 125.0 119.6 120.9 121.9 120.3 121.9 120.7 123.1 119.6 121.6 119.5 122.1 121.5 121.5 119.6 119.5 120.4 120.0 117.4 122.3 119.5 120.0 115.3 118.6 120.3 122.2 120.7 117.9 121.2 115.3 118.6 120.3 120.3 120.7 120.0 120.0

RMSD value for Bond Length B3LYP/6-311G is 0.019.

RMSD value of Bond Angle B3LYP/6-311G is 1.303.

### 3.2 Spectral studies

Based on UV (Figure S1) spectral studies, the transistions are observed theoretical value as same as the experimental value. The band values are 334 nm and 294 nm are corresponding to n- π∗ transitions; and π-π∗ transitions. The IR (Figures S2 and S5) and NMR (Figures S3 and S4) spectra, the calculated and theoretical values are given in Tables 3 and 4.

**Table 3:** Comparison of the observed and calculated vibrational spectra of the title compound.

Assignments	Theoretical frequencies B3LYP/6311G	Observed frequencies (FTIR)
C=N	1644	1637
OH	3436	3434
C-H	2956	2956
C=C	1652	1654
**RMSD**	**3.77**

**Table 4:** Theoretical and experimental ^13^C and ^1^H isotropic chemical shifts [with respect to TMS, all values in ppm] for Schiff base molecule.

Assignments	^1^H-NMR		Assignments	^13^C-NMR	
	Experimental (δ = ppm)	Theoretical B3LYP/6311G		Experimental (δ = ppm)	Theoretical B3LYP/6311G
1H s, OH	3.14	2.8	HC = N, C7	168.10	166.86
1H, s, -CH = N	8.56	7.66	Ar-OCH_3,_ C1	150.47	155.06
m, Ar–H	6.9–7.21	5.64–5.99	Ar- OH, C2	148.30	152.13
3H, s, O-CH_3_	3.82	2.8	Ar–N=C, C8	145.88	146.13
3H, s, CH_3_	2.24	1.6	O-CH_3_, C17	56.31	52.55
6H, s, CH_3_	2.10	1.5	-CH_3,_ C15	18.47	14.66
			-CH_3_ C14,C16	20.85	15.47
			Ar–C, C4-C6, C9-C13	119.95–134.15	118.75–135.63
**RMSD**		**0.9111**	**RMSD**		**3.3093**

### 3.3 Hirshfeld surface analysis

**Figure 3:**
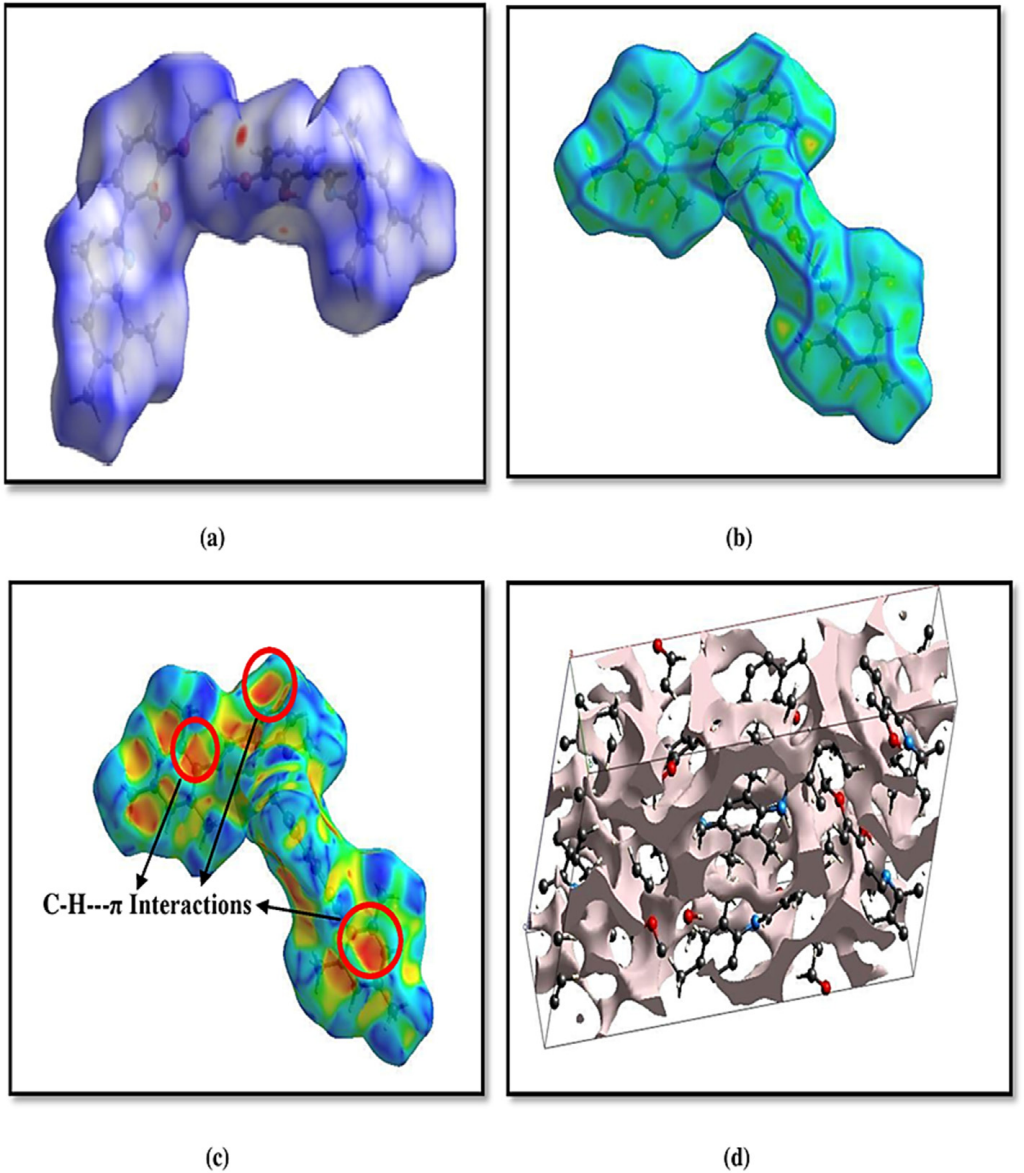
(a) The Hirshfeld surface plots over d _norm._ (b) Hirshfeld surface mapped over the curvedness (c) Hirshfeld surface mapped over the shape index (d) Crystal void plots.

The intermolecular interactions in the title compound (I), the *d*_*norm*_, is in the range -0.1350 to 1.3583 a.u. The crystal-void calculation (using 0.002 a.u. as the iso value) shows (see Figure 3a & d) that the volume of the void in the compound is 479.57 Å^3^ and the surface area is 1382.07 Å^2^.

Using porosity as a factor, the calculated void volume of the compound is 18%.The red spots on the Hirshfeld surface represent N-H…O contacts while the blue regions correspond to weak interactions and white spots are due to H…H contacts. When the Hirshfeld surface is mapped onto further properties such as curvedness, shape index (see Figure 3b and c) it provides additional insight into the crystal packing and into the intermolecular interactions of crystal structures. Shape index curves appear complementary red (pits) and blue (bumps), which represent negative and positive surface property values. In the crystal structure, three C-H ···π interactions show up on the Hirshfeld surface mapped over the shape index (range −1.0 0 0 to + 1.0 0 0 a.u).

**Figure 4:**
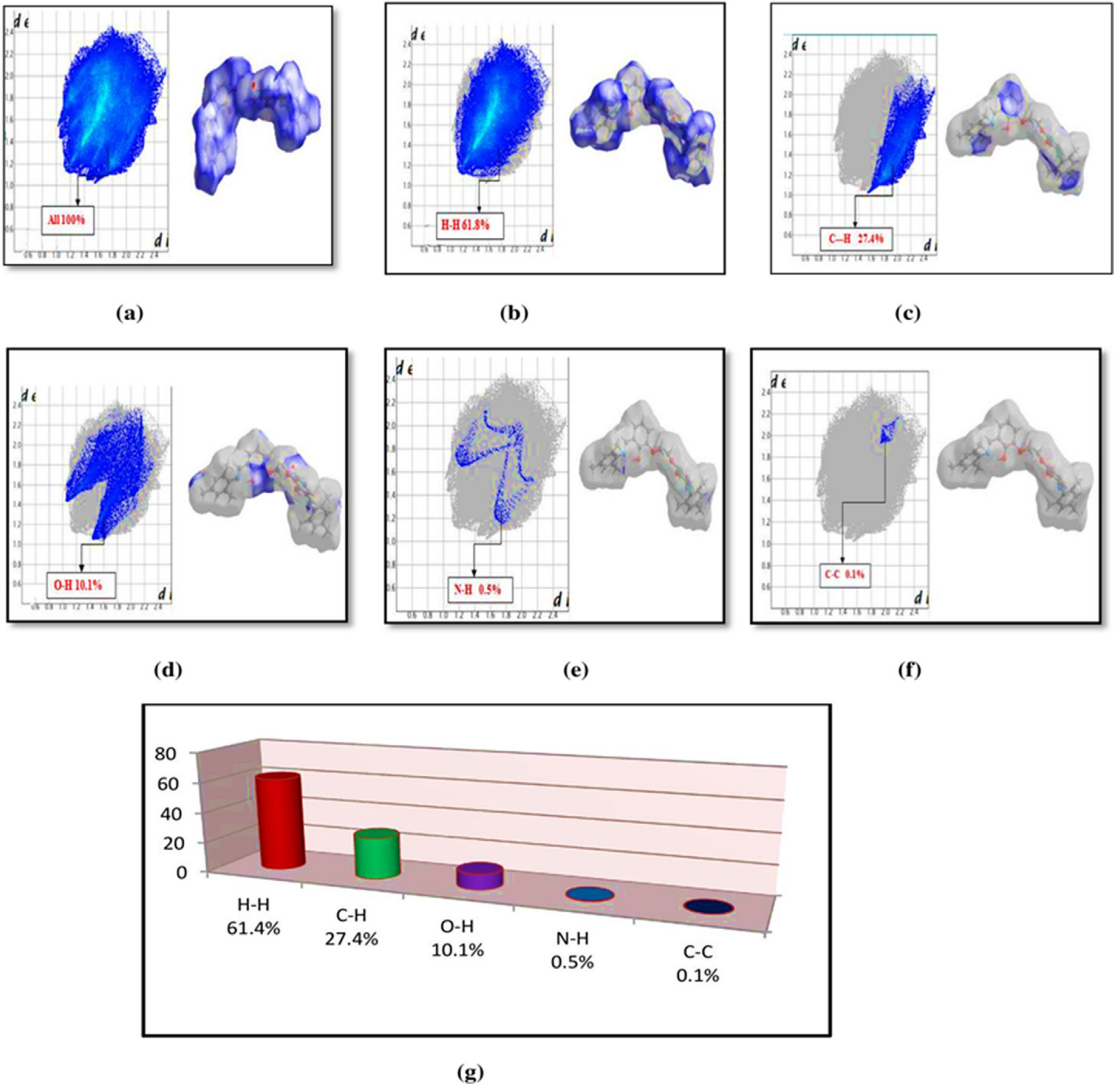
Two-dimensional fingerprint plot for the compound (I) showing the contributions of individual types of interactions: (a) All intermolecular contacts, (b) H--- H contacts, (c) C---H/H---C contacts, (d) H---O/O---H contacts, (e)N---H/H---N contacts (f) C---C/C---C contacts (g) Relative percentage contributions of close contacts to the Hirshfeld surfaces.

Two-dimensional finger print plots from Hirshfeld surface analysis (see Figure 4). H…H (61.4 %) contacts make the largest contribution to the Hirshfeld surfaces and give a break-down of different contacts as follows: O…H/H…O (10.1 %), C…C (0.1 %), C…H/H…C (27.4 %), N…H/H…N (0.5 %).

### 3.4 Hydrogen bonding interactions

#### 3.4.1 Interaction energies between molecular pair

The inter-molecular interactions were analyzed by Gavezzotti's PIXEL method [33, 34, 35, 36, 37] for the selected molecular pair and also by the Crystal Explorer model [20]. The calculated total interaction energy from the above-mentioned methods is the sum of electrostatic, E_elec_, Polarisation, E_pol_, dispersion, E_disp_ and repulsion, E_rep_ energies as expressed in Eq. (1).

(1) E_tot_ = E_elec_ + E_pol_ + E_disp_ + E_rep_

It is observed that the crystal packing is highly stabilized by dispersion energy, E_disp_; -144.9 Kcal/mol. The interaction energies between the molecular pairs of different components calculated from both PIXEL and Crystal Explorer models were listed see Table 5.

**Table 5:** PIXEL energy for experimental and predicted crystal structures and between the molecular pairs (Kcal/mol).

Bond	Symmetry	Distance	E_elec_	E_pol_	E_disp_	E_rep_	E_tot_
C17B-H73B...O2A	x,y,z	11.152	-9.8/-8.5	-3.2/-2.7	-9.6/-9.8	7.6/7.7	-15.1/-14.8
C12A-H12A...O1A;C12A-H12A...C2A;C14A-H43A...N1A	-x+1,+y-1/2,-z+1/2	5.977	-20.3/-14.0	-10.7/-2.4	-60.1/-60.6	50.4/39.4	-40.7/-45.0
C6A -H6A...O1B;C6A -H6A...C2B	x,-y+1/2,+z-1/2	8.493	-12.4/-11.6	-5.3/-3.3	-30.2/-29.9	22.1/15.6	-25.8/-28.3
C12B-H12B...O1B	-x+2,+y+1/2,-z+1/2 + 1	6.764	-11.9/-9.9	-6.5/-2.2	-47.5/-50.3	30.8/30.3	-35.1/-37.2
C6B-H6B...O1A; C6B-H6B...C2A	x,+y+1,+z	9.073	-9.8/-10	-5.1/-3.7	-28.2/-28.1	18.8/13.5	-24.3/-27
Crystal Structure			-41.5	-19.4	-144.9	87.2	-118.6

Among the molecular pair interactions see Table 6, the total interaction energy, E_tot_ for the molecule at -x+1, +y-1/2, -z+1/2 accounting for the bifurcated hydrogen bond C12A-H12A...O1 and C12A-H12A...C2A is found to be high and the values were -40.7 Kcal/mol [PIXEL] and -45.0 Kcal/mol [Crystal Explorer]. The interaction energies with the molecule framing the other two bifurcated H-bonds; C6A-H6A...O1B/C6A -H6A...C2B and C6B-H6B...O1A/C6B-H6B...C2A were -25.8 Kcal/mol [PIXEL], -28.3 [Crystal Explorer] and -24.3 [PIXEL], -27.0 [Crystal Explorer]. Despite the bifurcated H-bonds, the stability of the crystal packing is further enhanced by C12B-H12B...O1B, H-bond interactions with E_tot_ values -35.1 Kcal/mol and -37.2 Kcal/mol calculated from both the models.

**Table 6:** Intra and Inter molecular hydrogen bond interactions.

H-bond	D…A	H…A	D-H…A
O1A-H1A...N1A∗	2.604	1.881	146.4
O1B-H2B...N1B∗	2.601	1.876	146.7
C17B-H73B...O2A∗	3.330	2.597	133.4
C12A-H12A...O1A^1^	3.462	2.671	143.3
C12A-H12A...C2A^1^	3.586	2.884	133.3
C14A-H43A...N1A^1^	3.655	2.973	129.2
C6A -H6A...O1B^2^	3.542	2.652	160.6
C6A -H6A...C2B^2^	3.529	2.828	133.1
C12B-H12B...O1B^3^	3.559	2.789	140.9
C6B-H6B...O1A^4^	3.593	2.803	143.4
C6B-H6B...C2A^4^	3.615	2.732	158.8

#### 3.4.2 Closed-shell interactions

The QTAIM analysis was carried out for investigating the strength of intra and inter-molecular H-bond interactions. The topological parameters of important interactions calculated at (3,-1) bond critical point on the bond path (see Table 7). From this table, it was calculated that the ratio of V(r); the potential energy, and G(r); the kinetic energy density is ∼1 which attributes to the closed-shell nature of interactions. As expected, O-H…N intra molecular interactions are the strongest non-covalent interactions, as their average energy value is -37.8 KJ/mol. Among the three bifurcated H-bond [C-H…O & C-H…C] interactions, the interaction energies of two bifurcated H-bond; C12A-H12A…O1A/C2A and C6A-H6A…O1B/C2B is ∼ -5.7 KJ/mol which is slightly higher when compared with that of C6B-H6B…O1A/C2A bifurcated H-bond, whose energy value is -4.06 KJ/mol. Following the other interactions, C14A-H43A…N1A and C12B-H12B…O1B exhibits weak characters as their energy value is ∼ -3.0 KJ/mol.

**Table 7:** Topological properties of H-bond interactions at the (3, -1) bond critical points.

S.No	H-bond	Ρ e/Å^−3^	Δ^2^ρ e/Å^−5^	G(r) (KJ/mol)	V(r) (KJ/mol)	H(r) (KJ/mol)	E (KJ/mol)
1.	O1A-H1A...N1A∗	0.240	2.720	74.79	-75.53	-0.74	-37.76
2.	O1B-H2B...N1B∗	0.240	2.752	75.40	-75.87	-0.47	-37.93
3.	C17B-H73B...O2A∗	0.048	0.671	15.45	-12.62	2.83	-6.31
4.	C12A-H12A...O1A^1^	0.045	0.593	13.66	-11.16	2.50	-5.58
5.	C12A-H12A...C2A^1^	0.045	0.593	13.66	-11.16	2.50	-5.58
6.	C14A-H43A...N1A^1^	0.028	0.368	7.95	-5.87	2.07	-2.94
7.	C6A -H6A...O1B^2^	0.046	0.578	13.67	-11.60	2.06	-5.80
8.	C6A -H6A...C2B^2^	0.046	0.578	13.67	-11.60	2.06	-5.80
9.	C12B-H12B...O1B^3^	0.029	0.430	9.10	-6.49	2.61	-3.24
10.	C6B-H6B...O1A^4^	0.040	0.539	11.40	-8.13	3.27	-4.06
11.	C6B-H6B...C2A^4^	0.040	0.539	11.40	-8.13	3.27	-4.06

### 3.5 Frontier molecular orbital calculation (FMO)

**Figure 5:**
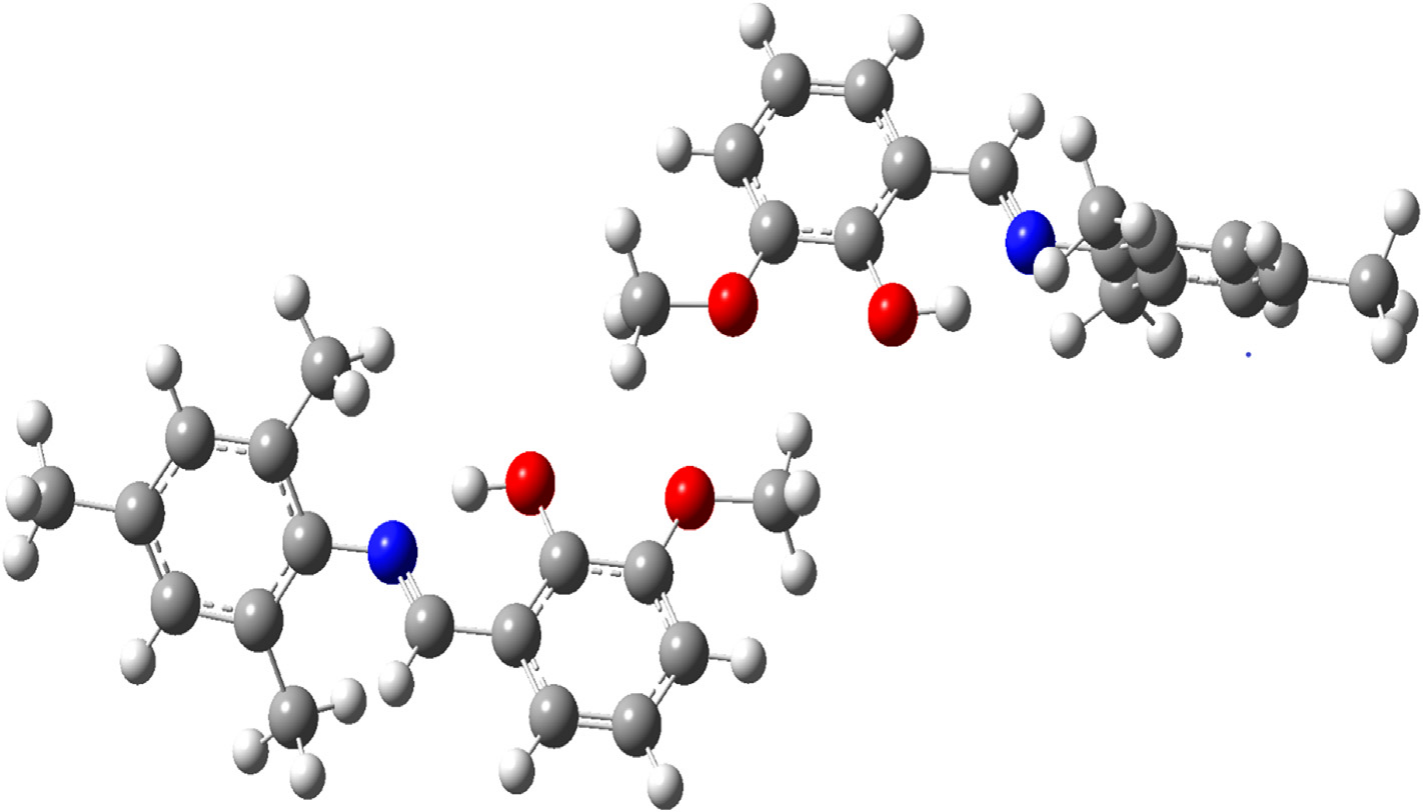
DFT Optimized structure of compound (I) (Modified figure).

In the present study, optimized Schiff base compound I was computed by B3LYP method with the 6-311G basis set (see Figure 5). The FMO energies (E_HOMO_, E_LUMO_) were used to calculate the global chemical reactivity descriptors of the title compound I such as the ionization potential (Ip), electron affinity (E_A_), the energy gap (E_LUMO_− E_HOMO_gap), the global hardness(η), the global softness(σ), the chemical potential(μ), the electronegativity(χ) and the electrophilicity index(ω) [38, 39, 40, 41, 42, 43]. These important descriptors are calculated by the following equations.

Ip = −E _HOMO_

E_A_ = −E _LUMO_

E_gap_ = (E_LUMO_ −E_HOMO_)

(η) = I –A/ 2

(σ) = 1/ η

(μ) = –I + A /2

(χ) = I + A/ 2

(ω) = μ^2^/ 2

**Figure 6:**
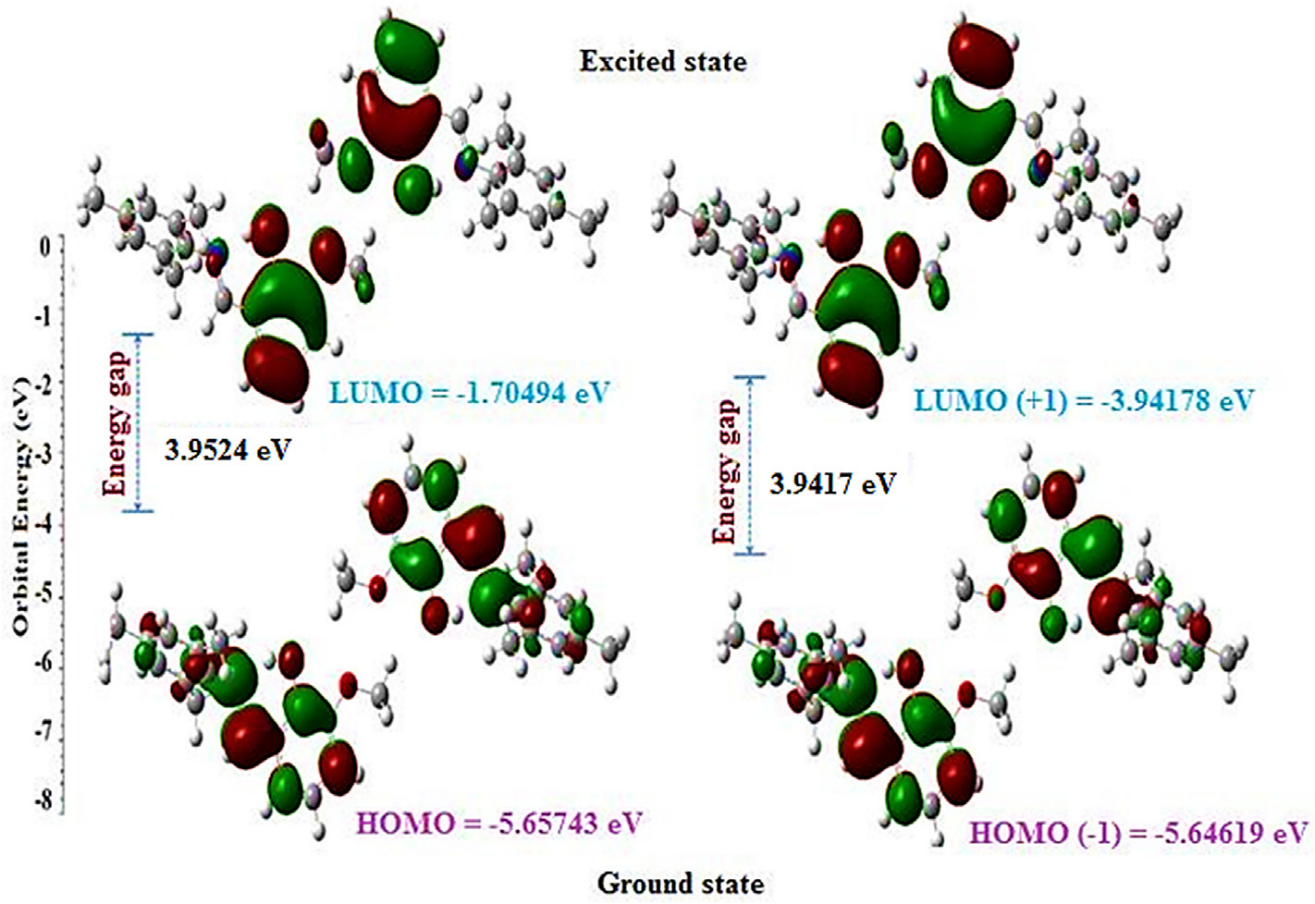
The graphical presentation of the highest occupied and lowest unoccupied molecular orbitals of compound (I).

Where A is the ionization potential (Ip) and I is the electron affinity (E_A_) of the molecule. Both parameters can be expressed through highest occupied molecular orbital and lowest unoccupied molecular orbital energies as Ip = –E_HOMO_ and E_A_ = –E_LUMO_. The calculated values of the Ionization potential, electron affinity, the energy gap, the global hardness, the global softness, the chemical potential, the electronegativity and the electrophilicity index of the molecule (see Table 8) and the values are 5.6574, 1.7049, 3.9525, 1.9762, 0.5060, 3.6811, -3.6811, 3.4285eV respectively (see Figure 6).

**Table 8:** Calculated frontier molecular orbital analysis of the compound (I).

Parameters	Values (eV)
E_HOMO_	-5.6574
E_LUMO_	-1.7049
E_HOMO-1_	-5.6462
E_LUMO+1_	-1.7044
E_LUMO_− E_HOMO_ gap	3.9525
E_LUMO+1_– E_HOMO-1_ gap	3.9418
Chemical hardness(η)	1.9762
Softness (σ)	0.5060
Chemical potential (μ)	3.6811
Electronegativity (χ)	-3.6811
Electrophilicity index (ω)	3.4285

### 3.6 Biological studies

**Figure 7:**
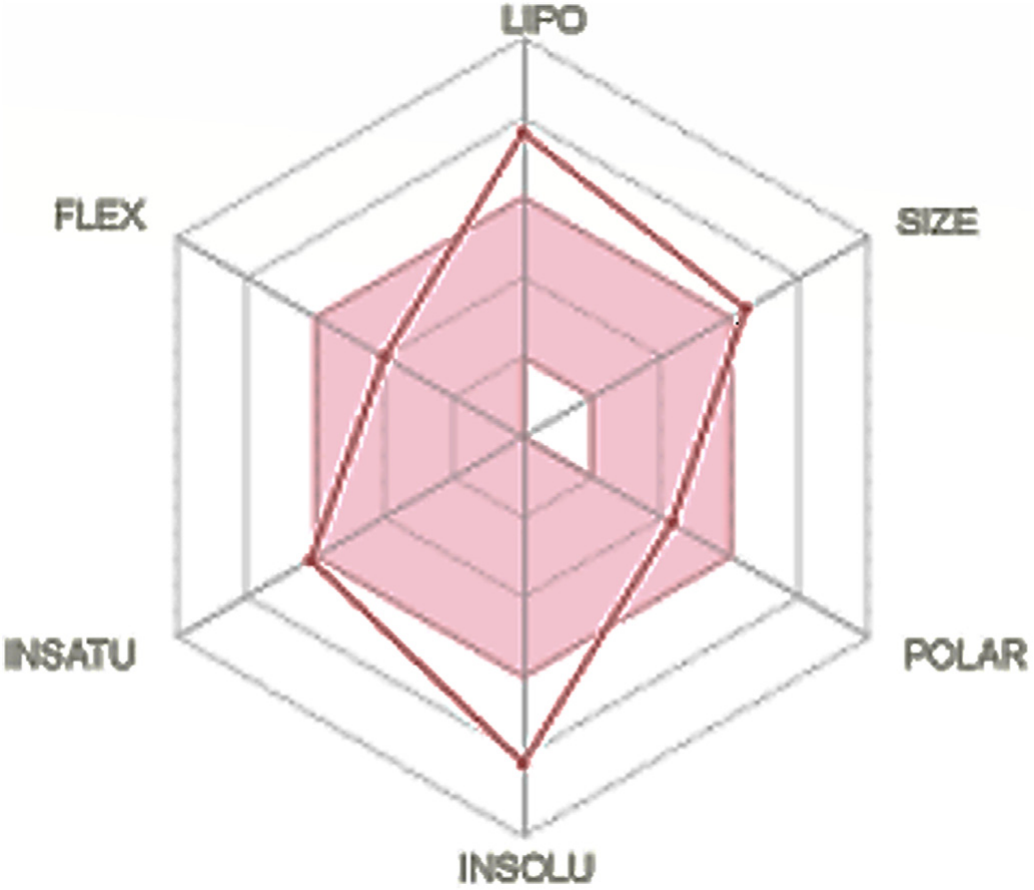
Bioavailability radar chart of compound (I). [Pink area in plotted graph represents a favourable set of properties for excellent oral bioavailability. LIPO (Lipophilicity), XLOGP between −0.7 and +5.0, SIZE (Molecular weight and range = 150–500 g/mol), POLAR (Polarity), TPSA (20 and 130), INSOLU (Solubility), LogS not higher than 6, INSATU (Saturation), FLEX (Flexibility), and no more than 10 rotable bond].

In-silico ADME (Absorption, Distribution, Metabolism and Excretion) study of the Schiff base compound (I) was performed and physiochemical properties were calculated by using Swiss ADME web tool. The bioavailability radar chart of the compound (I) (see Figure 7) and the values of RB, HBA, HBD, TPSA, LogP, LogS and other parameters (see Table 9).

**Table 9:** Physiochemical properties of compound (I).

Parameters	Values
RB	6
HBA	6
HBD	2
TPSA	83.64
BBB permeant/GI Absorption	No/Low
Log P	6.28
Log S	-5.72
Lipinski/violation	No/2
Water Solubility	Moderately Soluble
Vailability score	0.17

[The parameters are stand as such RB, number of rotatable bonds; HBA, number of hydrogen bond acceptors; HBD, number of hydrogen bond donors; TPSA, topological polar surface area; Log P, lipophilicity; Log S, water solubility; Lipinski violations, Bioavailability score].

### 3.7 Determination of minimum inhibitory concentration (MIC) of {3-Methoxy-6-[(2, 4, 6- trimethyl-phenylamino)-methyl]-phenol}

**Figure 8:**
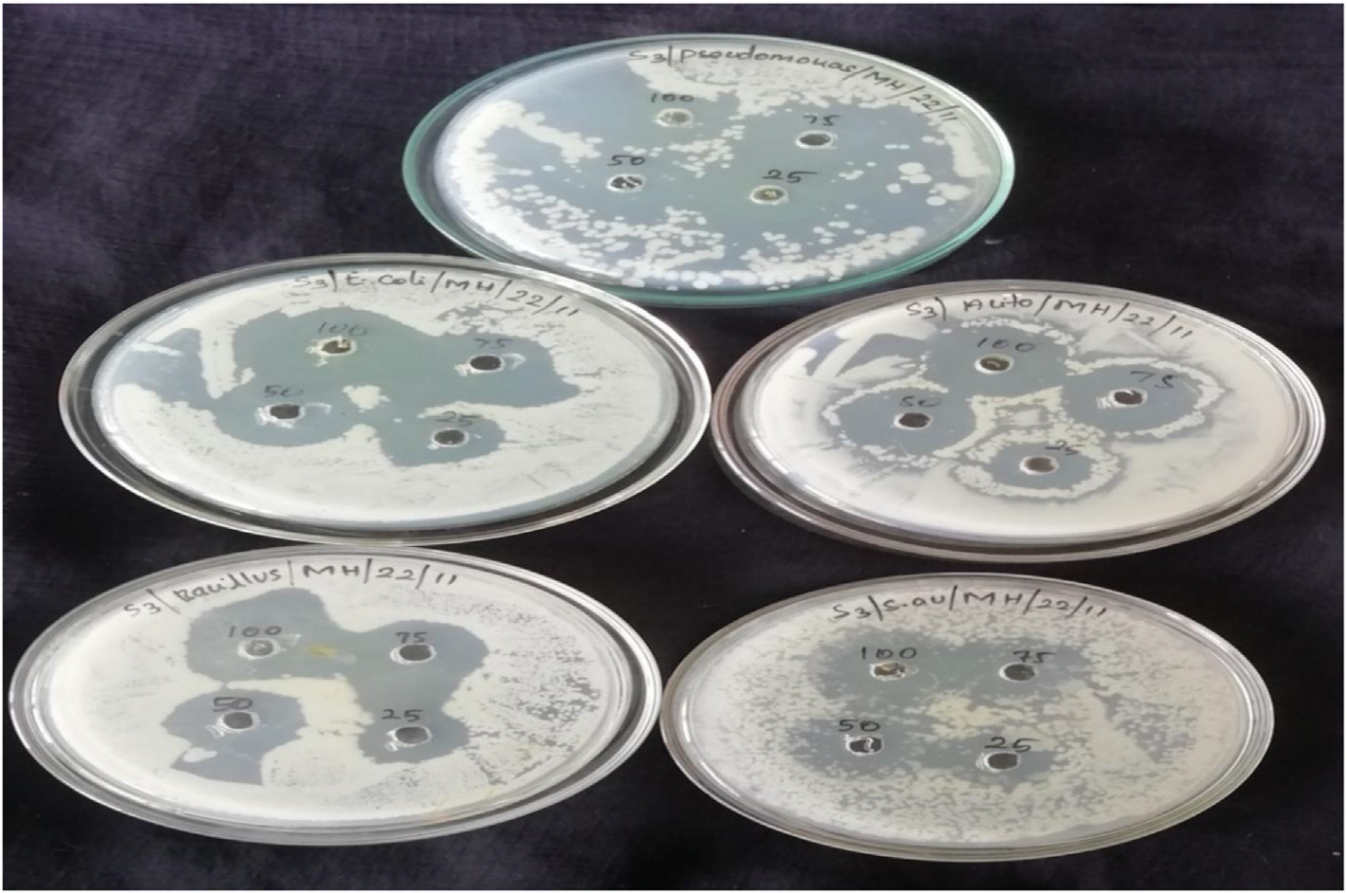
Antibacterial activity of Schiff base compound (I) against microorganisms.

**Figure 9:**
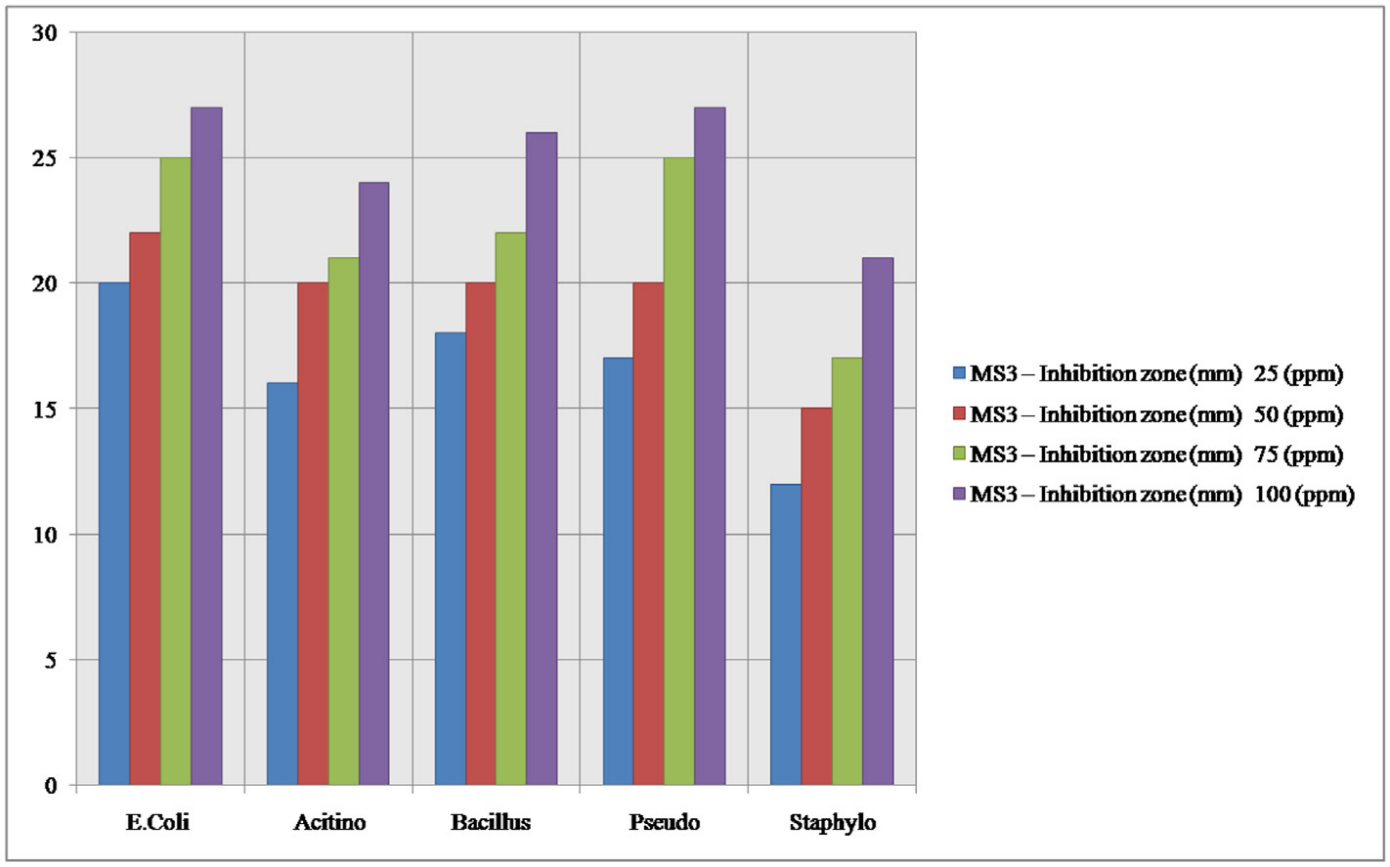
Bar graphs showing zone of inhibition introduced by Schiff base compound (I) against various microorganisms.

The sterilized Muller Hinton agar was poured into the five petri plates and solidified. And make 5-mm in diameter wells were cut from the agar using a sterile cork borer, and 20 μL of the {3-Methoxy-6-[(2, 4, 6- trimethyl-phenylamino)-methyl]-phenol} stock substances with different concentrations viz (25, 50, 75 and 100 ppm was delivered into the different wells. The plates were incubated for 24 h at 35 ± 2°Cin bacterial incubator. After 24 h the antimicrobial activity was analysed by measuring the radius of the inhibition zone against the test organism using a digital calliper. DMSO diluted to 1:10 was used as the solvent standard control to ensure that there is no bacterial growth. The Schiff base compound {3-Methoxy-6-[(2, 4, 6- trimethyl-phenylamino)-methyl]-phenol} was tested with different concentrations (25, 50, 75 and 100ppm; see Table 10) against *Escherichia coli, Actinobacteria, Bacillus Subtilis* and *Staphylococcus aureus and Pseudomonas aeruginosa*). The antibacterial study was revealed that the compound (I) showed excellent activity that inhibit the test organisms. The highest inhibition was observed at 100 ppm of each test organisms viz., *Staphylococcus aureus* (21mm), *Pseudomonas aeruginosa* (27mm), *Escherichia coli* (27mm), *Actinobacteria* (24 ​mm) and *Bacillus Subtilis* (26 ​mm) (see Figures 8 and 9).

**Table 10:** Antibacterial activity of compounds as the diameter of zone of inhibition (mm).

Organism	Inhibition zone (mm) - DMSO (Control)			
	25 (ppm)	50 (ppm)	75 (ppm)	100 (ppm)
*E.Coli*	20	22	25	27
*Acitinobacter*	16	20	21	24
*Bacillus*	18	20	22	26
*Pseudomonas*	17	20	25	27
*Staphylococcus aureus*	12	15	17	21

**Figure 10:**
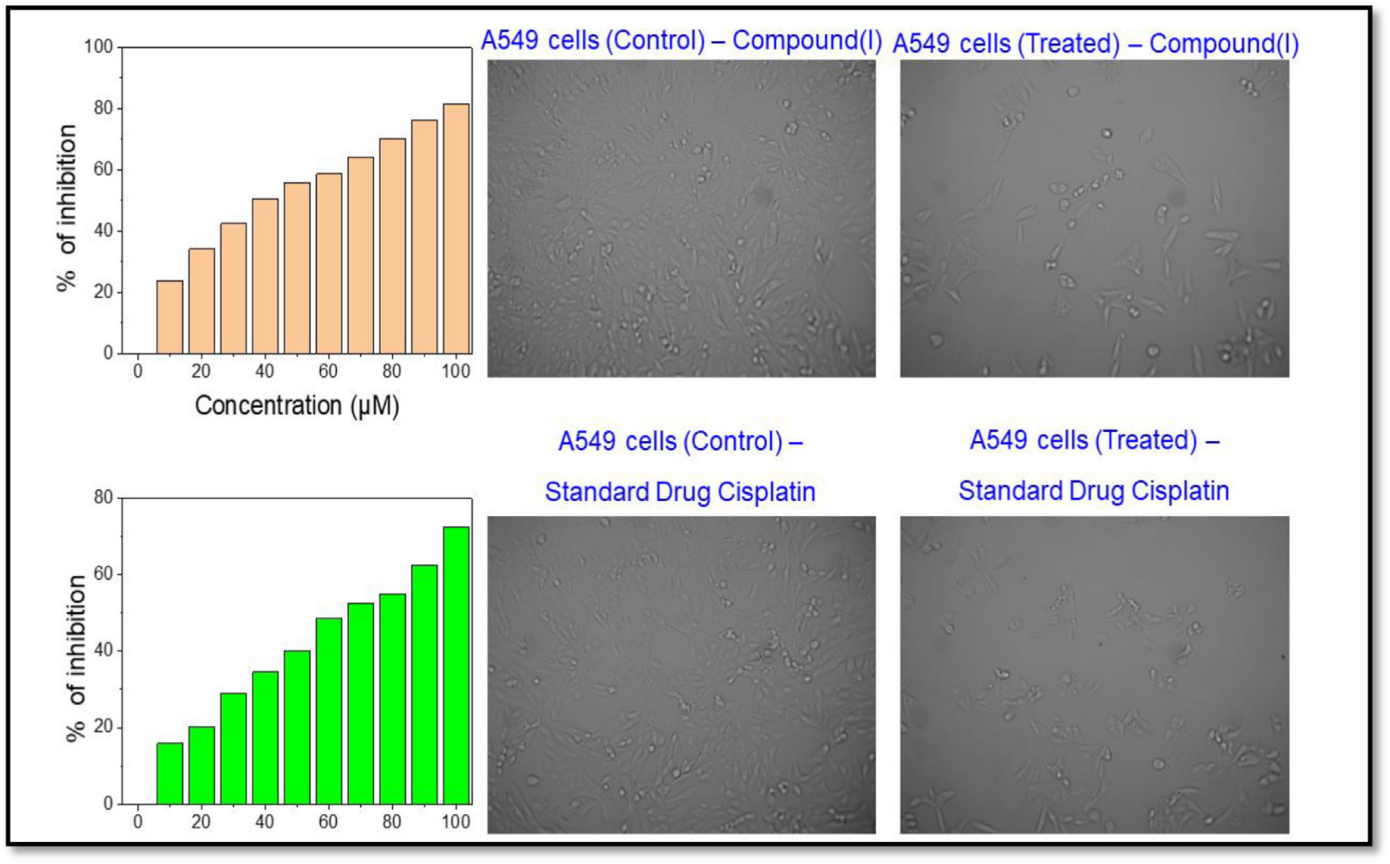
Cell proliferation was examined by MTT assay. Cytotoxicity with compound(I) and standard drug cisplatin for 24 h. Morphological observation of A549 cells after incubation with IC_50_ concentration of compound(I) and standard drug cisplatin for 24 h.

The in vitro cytotoxicity effects of compound (I) was evaluated against the human lung carcinoma (A549) cell line using the 3-(4,5-dimethylthiazol-2-yl)-2,5-diphenyltetrazolium bromide (MTT) assay at different concentrations (0–100 μM) for 24 h incubation. Results that were concentration-dependent showed that the relative viability of the cells decreased with increasing concentrations of the title compound. The title compound (I) showed higher activity in A549 (77.05 μM) cells, which was also higher than for the standard anticancer agent cisplatin (see Figure 10). After the A549 cells were exposed to various concentrations of compound (I) and cisplatin (standard drug) for 24 h, the compound(I) and cisplatin reduced cell viability in an IC_50_ concentration, as determined by microscopy [44].

### 3.8 Molecular docking of studies on anti-cancer activity of the MTP

The MTP compound is docked with the human dihydrofolate reductase (hDHFR) enzyme which is prominent drug target in the chemotherapy of cancer treatments. The function of hDHFR is to convert dihydrofolic acid into tetrahydrofolic acid by reduction reaction by the help of NADH as the electron donor. The crystal structure of NADP+ and folatecomplexedhDHFR is used for the molecular docking studies of MTP compound. When compared to other crystal structures of hDHFR the 4M6K structure was complexed with the enzyme substrate form and NADH+ with high resolution of 1.40 Å. For choosing the docking site for the MTP compound, a test molecular docking of MTP with hDHFR was carried out. The hDHFR has two sites for inhibiting the enzyme activity, they are the substrate folic acid binding site and the Co-factor binding site, the grid parameters chosen using the co-ordinates of NADP + binding site and folate binding site. Comparing the molecular docking of MTP with NADP+ and folate binding site, the MTP-NADP + region complex interacts with functionally important residues of that site with least binding energy of -7.94 kcal/mol. The important residues of NADP + binding site were ALA 9, ILE 16, ASP 21, LYS 54, LYS 55, THR 56, LEU 75, SER 76, ARG 77A, GLY 117, SER 118 [45].

**Figure 11:**
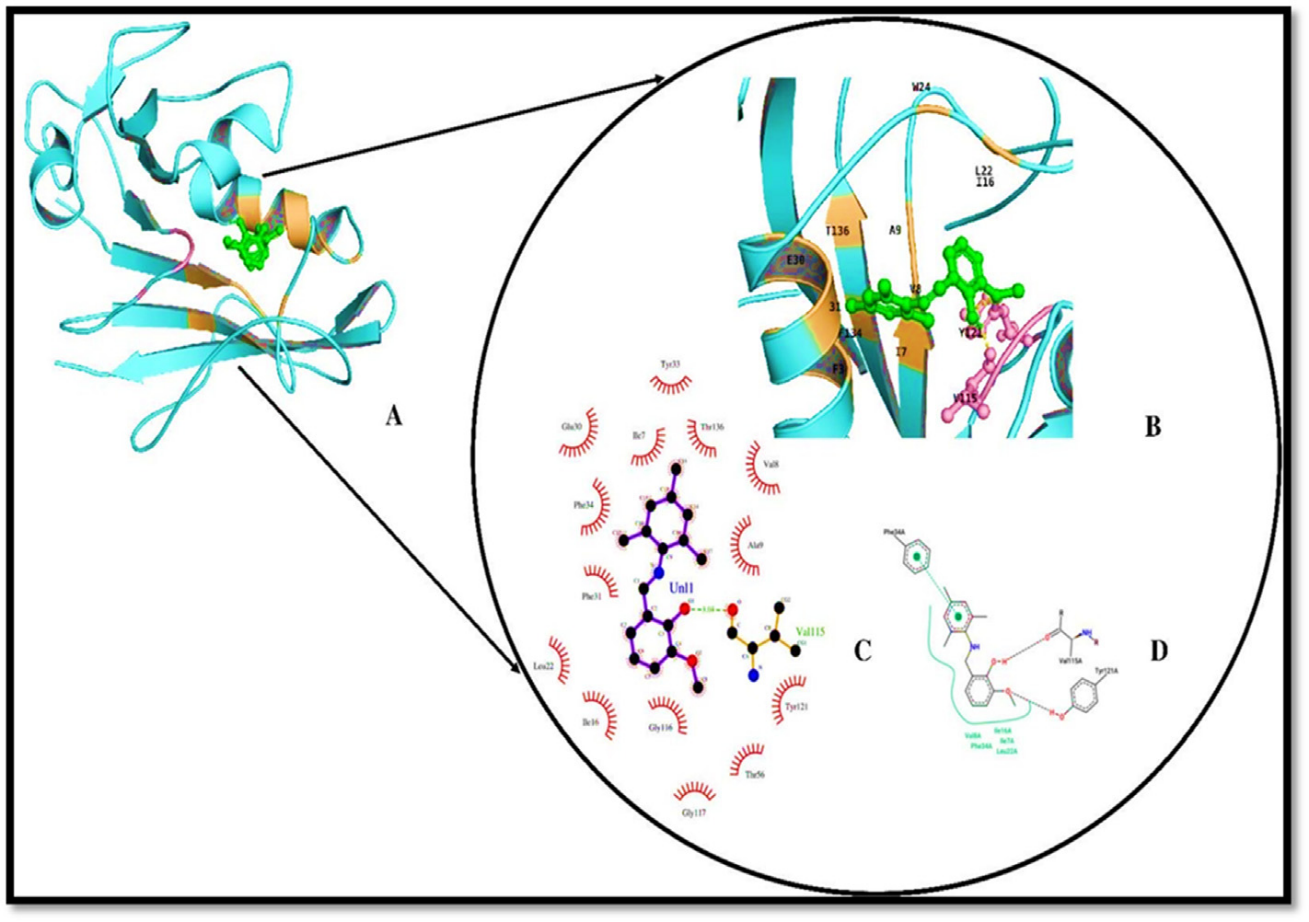
(A) Three-dimensional representation of compound bound DHFR (PDB:4M6K). DHFR (cyan cartoon), compound (Green stick), Hydrogen and Hydrophobic interacting regions (orange). (B) Hydrogen and Hydrophobic interactions (orange cartoon), compound (green stick), amino acid involved in hydrogen bond (pink stick). (C) 2-D image of ligand-receptor interaction generated using LIGPLOT+ (D) 2-D interaction image to analyse pi-pi interaction generated by poseview server Pi-Pi interaction (discontinued line connecting points from two rings).

The interaction of hDHFR resembles the interaction pattern of NADP+ with two hydrogen bonds on vanillin region to Val115 and Tyr 121 (see Figure 11A and B). The new pattern of interaction was obtained in the compound when compared to docking of compound with other two bacterial enzymes, the involvement of trimethyl phenyl region of the compound having pi-pi interaction with residue PHE 34 in the NADP binding site (see Figure 11C). Analyzing the overall hydrophobic interaction pattern, the vanillin region and trimethyl phenyl groups equally involves in hydrophobic interaction with residues CYS 6 to TYR 33 (see Figure 11D & Table 11).

**Table 11:** Hydrogen and Hydrophobic interaction data.

PDB ID	Name	Hydrogen Bonds			Hydrophobic Interaction
		Donor	Acceptor	Bond Length	
4m6k	Human dihydrofolate reductase	MTP O1-H	O-C(VAL 115)	2.1 Å	VAL 84, PHE 34, **ILE 16**, ILE 7, LEU 22
		TYR 121 O-H	O-C (MTP)	3.0 Å	
1hnj	E.coli beta-ketoacyl-acp synthase iii	ASN 247 N-H	O-C (MTP)	3.2 Å	PHE 304, ILE 250, HIS 244, ALA 246, PHE 213, VAL 212

#### 3.8.1 Molecular docking of studies on Anti-Bacterial Activity of the MTP

The enzyme β-ketoacyl-[acyl-carrier-protein] synthase III (FabH) is an important enzyme specific to plant and bacteria for its survival and pathogenesis. This enzyme is a transferase class of enzyme involved in fatty acid synthesis pathway where its converts acetyl-CoA and malonyl-[acyl-carrier-protein] into acetoacetyl-[acyl carrier protein]. Inhibiting this enzyme leads to hindered bacterial cell wall synthesis pathway and pathogen host interacting phenomenon. Since this enzyme is a important target for antibacterial compounds against organisms such as *E. coli*, *Bacillus subtilis*, *Pseudomonas aeruginosa*, *Acinetobacter baumannii*, *Staphylococcus aureus*. The enzyme has its active site as catalytic triad of CYS 112, HIS 244, ASN 274 [[46], [47]].

**Figure 12:**
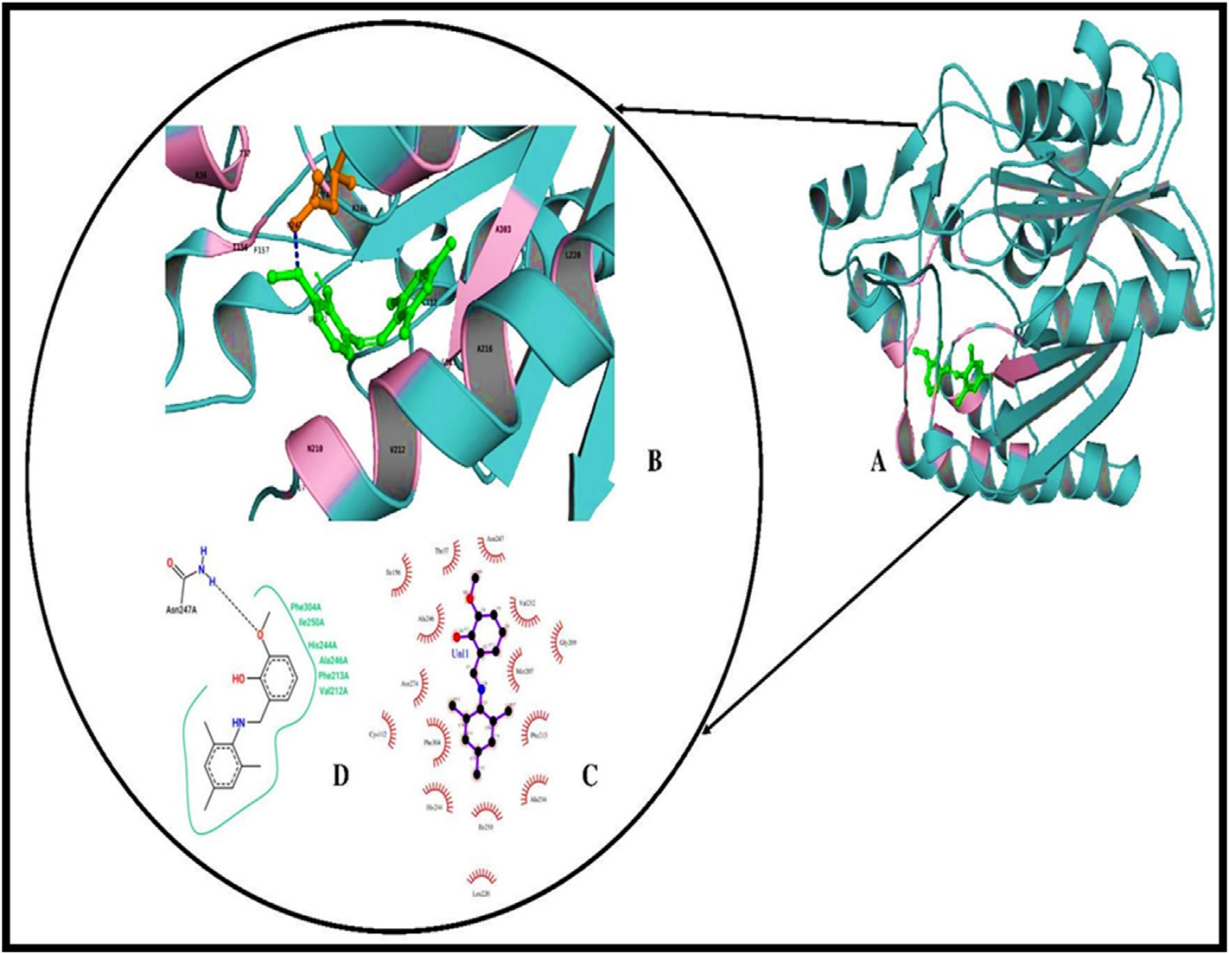
(A) Three-dimensional cartoon Representation of compound docked E. coli beta-ketoacyl-acp synthase iii (FabH) (PDB ID:1HNJ). Compound (green stick), FabH (dark cyan cartoon), Hydrogen and hydrophobic interacting regions (pink). (B) Three -dimensional figure of compound interacting with FabH. compound (green stick), Hydrogen bond interaction (orange stick) and Hydrophobic interacting regions (pink cartoon) (C) 2-D interaction image generated using LIGPLOT+ (D) 2D interaction diagram of FabH and compound prepared by poseview showing hydrogen bonds in black dotted line and hydrophobic interactions in continuous curved line.

The results from molecular docking of MTP compound in substrate binding area of Beta-keto-acyl carrier protein synthase iii (FabH) (PDB:1HNJ) gives ten stable poses having binding energy from 5.07 kcal/mol to -7.02 kcal/mol. The interaction analysis of ligand receptor complex shows that the oxygen group of vanillin region in the ligand interacts with ASN 247 of active site region through hydrogen bonding. The important catalytic residue HIS 244 of the FabH, makes hydrophobic interaction with the target compound. The complex has least binding energy of -5.36 kcal/mol (see Figure 12A to D & Table 12).

**Table 12:** Binding energy.

PDB ID	Name	Binding Energy (KCal/Mol)	Inhibition Constant (μM)	Intermolecular Energy (KCal/Mol)	VDW Desolv Energy (KCal/Mol)
4m6k	Human dihydrofolate reductase	-7.94	1.5	-9.14	-9.07
1hnj	E.coli beta-ketoacyl-acp synthase iii	-7.02	7.17	-8.12	-8.18

From the docking results its evident that the trimethyl phenyl moiety of the compound anticancer activity due to the presence of one pi-pi interaction with PHE 34 and one hydrogen bond and the antibacterial property of MTP compound is due to the vanillin moiety.

#### 3.8.2 Interactions with amino acid residues

**Figure 13:**
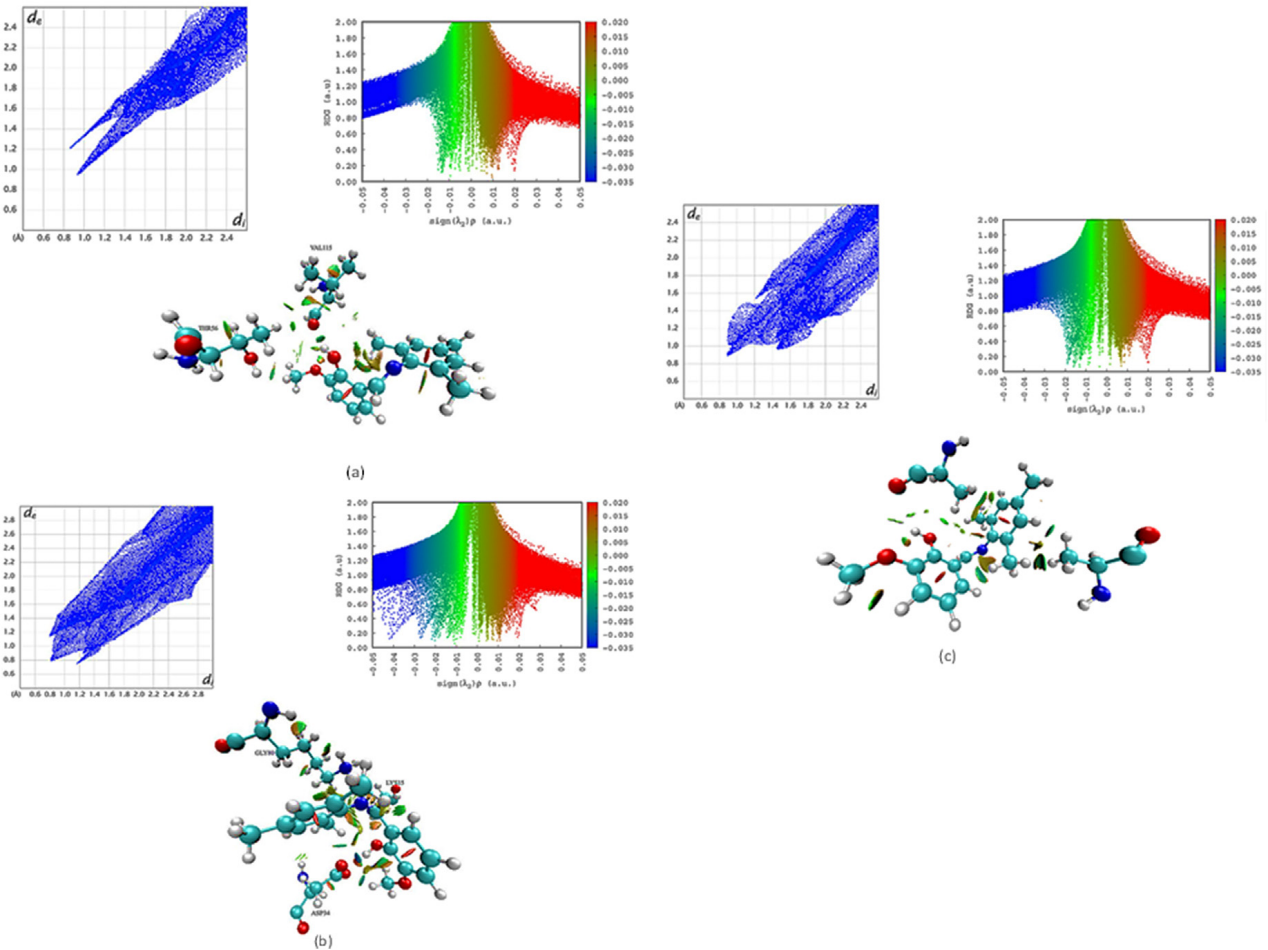
The Hirshfeld fingerprint map, RDG scatter and NCI iso-surface plot of MTP conformer in the active sites (a) 4BQS, (b) 4M6K and (c) 1HNJ proteins.

The affinity of MTP conformers towards the amino acid residues in the active site of 1HNJ, 4BQS, and 4M6K enzymes was well understood from the topological properties calculated at (3, -1) bcp (see Table 13). Notably ρ_cp_(r) [0.28 eÅ^−3^] and Δ^2^ρ_cp_(r) [1.67 eÅ^−5^] of N…N/LYS15 of MTP/4BQS complex is found to be the strongest with the energy value of -33.95 Kj/mol, among the three complexes. In MTP/4M6K complex, OH…O/VAL115 is found to have maximum binding energy [−15.47 KJ/mol] and MTP: OH…ALA246:O interaction in MTP/1HNJ complex has the least energy [−2.78 KJ/mol]. To quantify the ligand interactions with amino acid residues, Hirshfeld fingerprint maps (see Figure 13 a-c) were plotted for all three complexes. The sharp spikes in the fingerprint map confirm the strong H-bond interactions between MTP and the respective enzymes.

**Table 13:** Topological properties of H-bond interactions of MTP in the active site of 1HNJ, 4BQS and 4M6K enzymes.

PDB ID	H-bonds	ρ e/Å^−3^	Δ^2^ρ e/Å^−5^	G(r) (Kj/mol)	V(r) (Kj/mol)	H(r) (Kj/mol)	E (Kj/mol)
4M6K	MTP: OH…VAL115:O	0.10	1.19	-30.93	31.65	0.72	-15.47
	MTP:C…THR56:OG1	0.09	1.15	-24.88	28.15	3.27	-12.44
1HNJ	MTP: OH…ALA246:O	0.02	0.39	-5.57	8.08	2.51	-2.78
4BQS	LYS15:NZ…MTP:N	0.28	1.67	-67.91	56.30	-11.60	-33.95
	GLY80: N…MTP:O	0.14	1.38	-41.12	39.34	-1.78	-20.56
	MTP:OH…ASP34:OD2	0.18	1.66	-53.78	49.56	-4.22	-26.89
	MTP:C…ASP34:OD1	0.14	1.55	-41.12	41.67	0.54	-20.56

A non-covalent interaction (NCI) which includes hydrogen bonding, hydrophobic, electrostatic, and van der Waals interactions play a vital role in the protein-ligand complex. Therefore, to understand the binding mechanism of MTP in the active sites of 1HNJ, 4BQS and 4M6K receptor, NCI analysis was carried out. These NCI interactions were visualized from the isosurface plot which clearly shows the weak and strong interactions especially, the interactions that occur between the guaiacol group and the respective amino acid residues. The reduced density gradient [RDG] plots in which blue, green and red regions reveal the hydrogen bonding, van der Waals, and steric effect interactions between protein and ligand. Thus, from QTAIM, Hirshfeld fingerprint map and NCI analysis, it is concluded that the MTP ligand shows a strong binding affinity towards the active site of 4BQS protein when compared with the other two proteins.

## 4 Conclusion

The Schiff base compound (I) Crystal structure was characterized by using the SCXRD method. The Spectroscopic FTIR, UV-Vis and NMR studies were carried out and compared with the theoretical values obtained by using B3LYP method with the 6-311G basis set. The Hirshfeld surface and Fingerprint analyses were analyzed. The calculations of HOMO-LUMO energy gaps show that the molecule has good stability. Molecular Docking, invitro antibacterial and anticancer studies were carried out to identify the binding activity of Schiff base compound (I). Also, the binding strength between the ligand and protein was well examined from QTAIM analysis and NCI investigation has been carried out to explore the non-covalent interactions between them.

## Declarations

### Author contribution statement

Suganya Murugan: Performed the experiments; Wrote the Paper.

Jayasudha Nehru, David Stephen Arputharaj, Anaglit Catherine Paul, Prasanth Gunasekaran, Necmi Dege, Emine Berrin ÇINAR, Abdullah G. Al-Sehemi: Analyzed and interpreted the data.

Kasthuri Balasubramani, Venkatachalam Rajakannan: Conceived and designed the experiments; Analyzed and interpreted the data.

Jose Kavitha Savaridasson, Madhukar Hemamalini: Conceived and designed the experiments; Analyzed and interpreted the data; Contributed reagents, materials, analysis tools or data.

### Funding statement

This work was supported by SERB-IRE (Ref. No. SIR/2022/000011), Research Centre for Advanced Materials Science, 10.13039/501100007446King Khalid University, Abha 61413, Saudi Arabia (RCAMS/KKU/p001-21) and by Mother Teresa Women's University Tamil Nadu, India.

### Data availability statement

Data included in article/supplementary material/referenced in article.

### Declaration of interests statement

The authors declare no conflict of interest.

### Additional information

No additional information is available for this paper.

## Appendix A Supplementary data

Supplementary material Supplementary material

cif cif
