# Supplementary material for: Synthesis of 3-Methoxy-6- [(2, 4, 6-trimethyl-phenylamino)-methyl]-phenol Schiff base characterized by spectral, in-silco and in-vitro studies
Source: Heliyon. 2022 Aug 5;8(8):e10070. doi: 10.1016/j.heliyon.2022.e10070 (PMC9396558; doi:10.1016/j.heliyon.2022.e10070)
Supplement: Supplementary material [file mmc1.docx]

*Checking for embedded fcf data in CIF ...*
*Found embedded fcf data in CIF. Extracting fcf data from uploaded CIF, please wait***. . .**

**checkCIF/PLATON (basic structural check)**

Structure factors have been supplied for datablock(s) shelx

THIS REPORT IS FOR GUIDANCE ONLY. IF USED AS PART OF A REVIEW PROCEDURE FOR PUBLICATION, IT SHOULD NOT REPLACE THE EXPERTISE OF AN EXPERIENCED CRYSTALLOGRAPHIC REFEREE.

No syntax errors found. [CIF dictionary](https://www.iucr.org/iucr-top/cif/cif_core/definitions/index.html)
Please wait while processing .... [Interpreting this report](https://journals.iucr.org/services/cif/checking/checkcifreport.html)

[Structure factor report](https://checkcif.iucr.org/7Urd9YRaxPfYP/021722070458468313091/ckf.html)

**Datablock: shelx**

| Bond precision: | C-C = 0.0041 A | Wavelength=0.71073 |
| --- | --- | --- |

| Cell: | a=25.9845(12) | b=7.3318(4) | c=16.3543(8) |
| --- | --- | --- | --- |
|  | alpha=90 | beta=100.713(4) | gamma=90 |
| Temperature: | 296 K |  |  |

|  | Calculated | Reported |
| --- | --- | --- |
| Volume | 3061.4(3) | 3061.4(3) |
| Space group | P 21/c | P 21/c |
| Hall group | -P 2ybc | -P 2ybc |
| Moiety formula | C17 H19 N O2 | C17 H20.50 N2 O |
| Sum formula | C17 H19 N O2 | C17 H20.50 N2 O |
| Mr | 269.33 | 268.85 |
| Dx,g cm-3 | 1.169 | 1.167 |
| Z | 8 | 8 |
| Mu (mm-1) | 0.076 | 0.073 |
| F000 | 1152.0 | 1156.0 |
| F000' | 1152.50 |  |
| h,k,lmax | 31,8,19 | 31,8,19 |
| Nref | 5479 | 5464 |
| Tmin,Tmax | 0.971,0.993 | 0.974,0.993 |
| Tmin' | 0.958 |  |

| Correction method= # Reported T Limits: Tmin=0.974 Tmax=0.993 AbsCorr = INTEGRATION |  |
| --- | --- |

| Data completeness= 0.997 | Theta(max)= 25.137 |
| --- | --- |

| R(reflections)= 0.0579( 2882) | wR2(reflections)= 0.1356( 5464) |
| --- | --- |
| \| S = 1.027 \| Npar= 371 \| \| --- \| --- \| |  |

The following ALERTS were generated. Each ALERT has the format

**test-name_ALERT_alert-type_alert-level**.

Click on the hyperlinks for more details of the test.


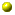
**Alert level C**

[PLAT041_ALERT_1_C](javascript:makeHelpWindow(%22PLAT041.html%22)) Calc. and Reported SumFormula Strings Differ Please Check

[PLAT043_ALERT_1_C](javascript:makeHelpWindow(%22PLAT043.html%22)) Calculated and Reported Mol. Weight Differ by .. 0.48 Check

[PLAT068_ALERT_1_C](javascript:makeHelpWindow(%22PLAT068.html%22)) Reported F000 Differs from Calcd (or Missing)... Please Check

[PLAT242_ALERT_2_C](javascript:makeHelpWindow(%22PLAT242.html%22)) Low 'MainMol' Ueq as Compared to Neighbors of O2A Check

[PLAT340_ALERT_3_C](javascript:makeHelpWindow(%22PLAT340.html%22)) Low Bond Precision on C-C Bonds ............... 0.00413 Ang.

[PLAT790_ALERT_4_C](javascript:makeHelpWindow(%22PLAT790.html%22)) Centre of Gravity not Within Unit Cell: Resd. # 1 Note

C17 H19 N O2

[PLAT906_ALERT_3_C](javascript:makeHelpWindow(%22PLAT906.html%22)) Large K Value in the Analysis of Variance ...... 49.196 Check

**And 2 other PLAT906 Alerts**

More ...

[PLAT911_ALERT_3_C](javascript:makeHelpWindow(%22PLAT911.html%22)) Missing FCF Refl Between Thmin & STh/L= 0.598 14 Report


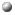
**Alert level G**

[FORMU01_ALERT_2_G](javascript:makeHelpWindow(%22FORMU_01.html%22)) There is a discrepancy between the atom counts in the

_chemical_formula_sum and the formula from the _atom_site* data.

Atom count from _chemical_formula_sum:C17 H20.5 N2 O1

Atom count from the _atom_site data: C17 H19 N1 O2

[CELLZ01_ALERT_1_G](javascript:makeHelpWindow(%22CELLZ_01.html%22)) Difference between formula and atom_site contents detected.

[CELLZ01_ALERT_1_G](javascript:makeHelpWindow(%22CELLZ_01.html%22)) ALERT: Large difference may be due to a

symmetry error - see SYMMG tests

From the CIF: _cell_formula_units_Z 8

From the CIF: _chemical_formula_sum C17 H20.50 N2 O

TEST: Compare cell contents of formula and atom_site data

atom Z*formula cif sites diff

C 136.00 136.00 0.00

H 164.00 152.00 12.00

N 16.00 8.00 8.00

O 8.00 16.00 -8.00

[PLAT007_ALERT_5_G](javascript:makeHelpWindow(%22PLAT007.html%22)) Number of Unrefined Donor-H Atoms .............. 2 Report

[PLAT042_ALERT_1_G](javascript:makeHelpWindow(%22PLAT042.html%22)) Calc. and Reported Moiety Formula Strings Differ Please Check

[PLAT066_ALERT_1_G](javascript:makeHelpWindow(%22PLAT066.html%22)) Predicted and Reported Tmin&Tmax Range Identical ? Check

[PLAT883_ALERT_1_G](javascript:makeHelpWindow(%22PLAT883.html%22)) No Info/Value for _atom_sites_solution_primary . Please Do !

[PLAT910_ALERT_3_G](javascript:makeHelpWindow(%22PLAT910.html%22)) Missing # of FCF Reflection(s) Below Theta(Min). 1 Note

[PLAT941_ALERT_3_G](javascript:makeHelpWindow(%22PLAT941.html%22)) Average HKL Measurement Multiplicity ........... 3.5 Low

[PLAT978_ALERT_2_G](javascript:makeHelpWindow(%22PLAT978.html%22)) Number C-C Bonds with Positive Residual Density. 0 Info

0 **ALERT level A** = Most likely a serious problem - resolve or explain

0 **ALERT level B** = A potentially serious problem, consider carefully

10 **ALERT level C** = Check. Ensure it is not caused by an omission or oversight

10 **ALERT level G** = General information/check it is not something unexpected

8 ALERT type 1 CIF construction/syntax error, inconsistent or missing data

3 ALERT type 2 Indicator that the structure model may be wrong or deficient

7 ALERT type 3 Indicator that the structure quality may be low

1 ALERT type 4 Improvement, methodology, query or suggestion

1 ALERT type 5 Informative message, check


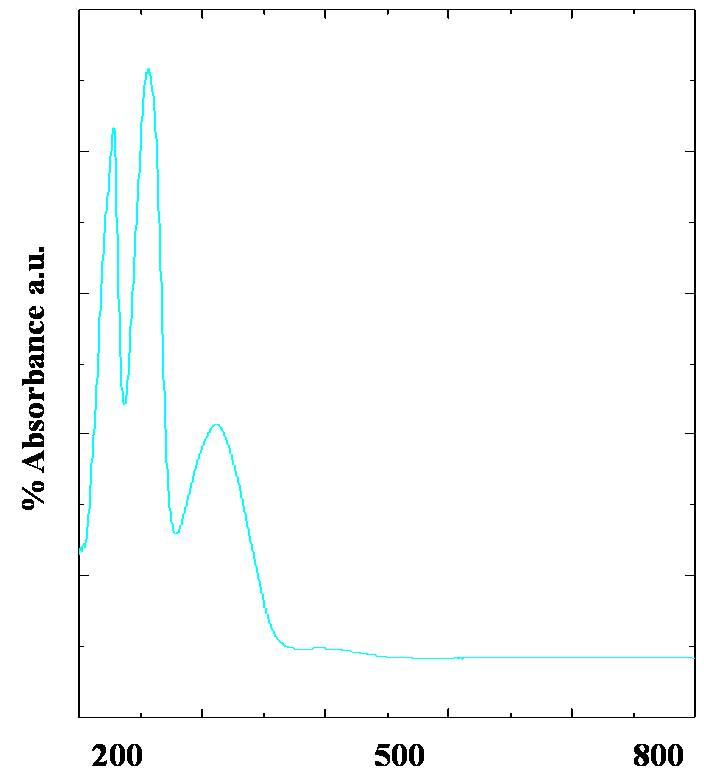


**Fig. S1.UV Spectrum of compound (I)**


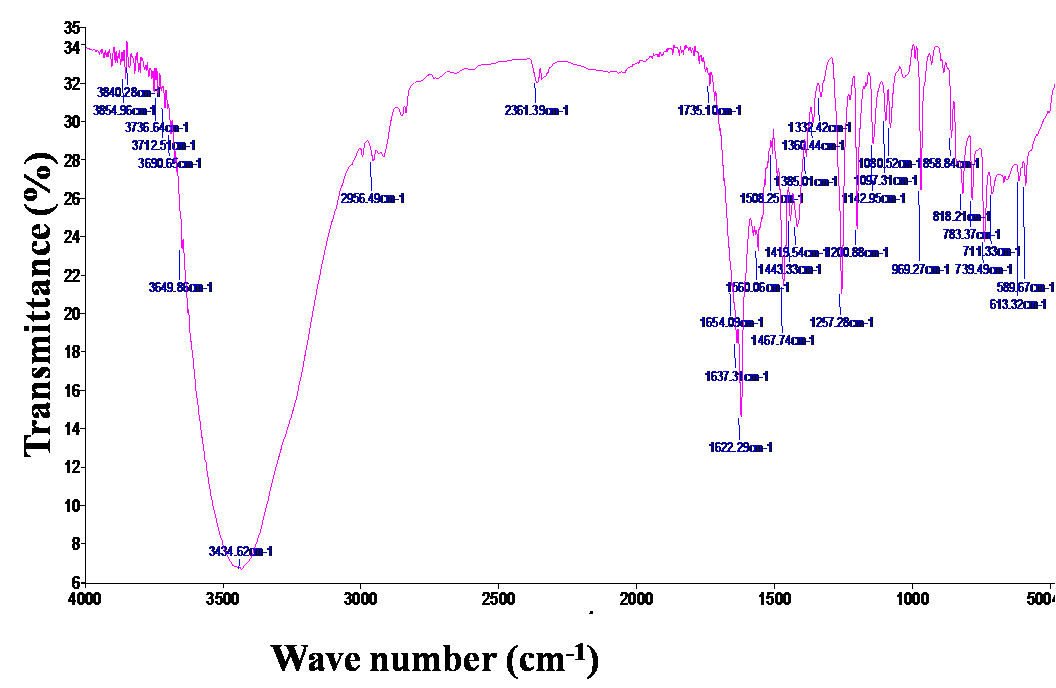


**Fig. S2: FT-IR Spectrum of compound (I)**


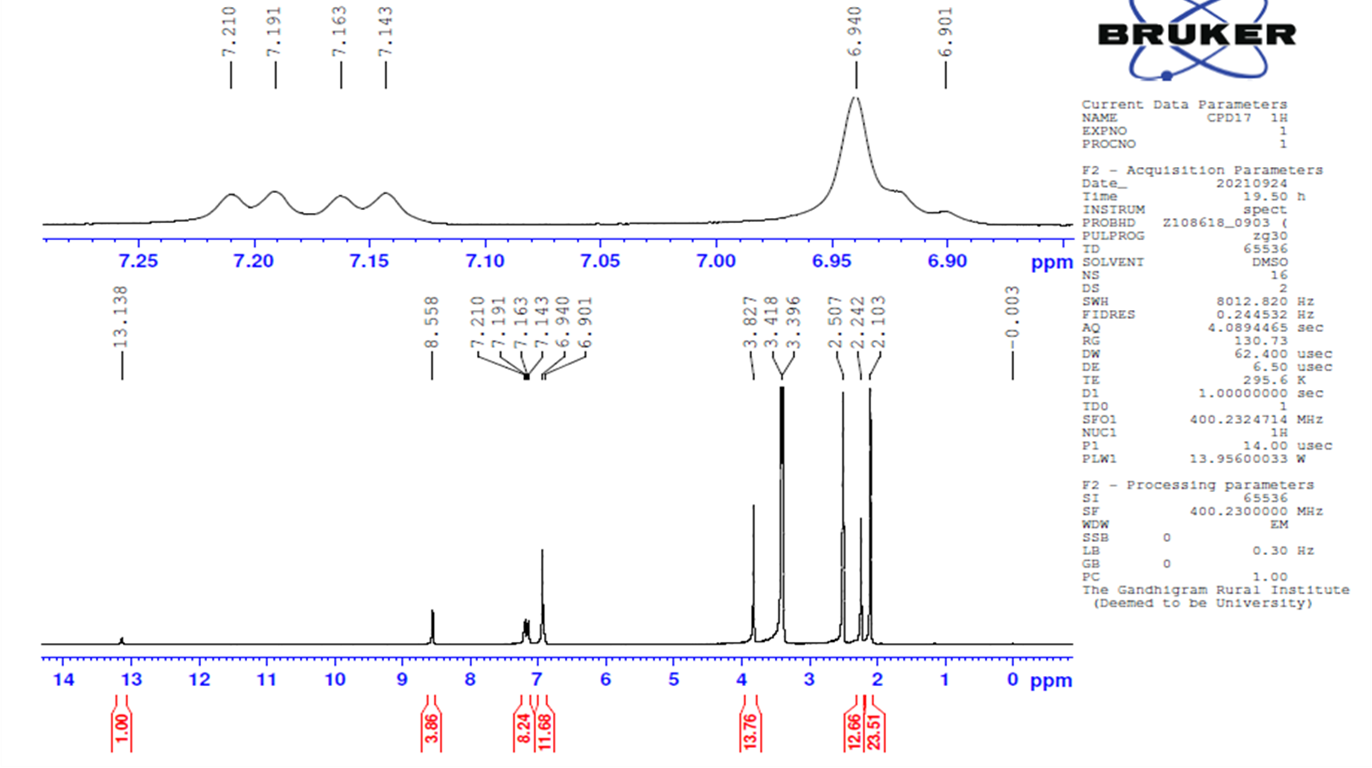


**Fig. S3:^1^H-NMR Spectrum of compound (I)**


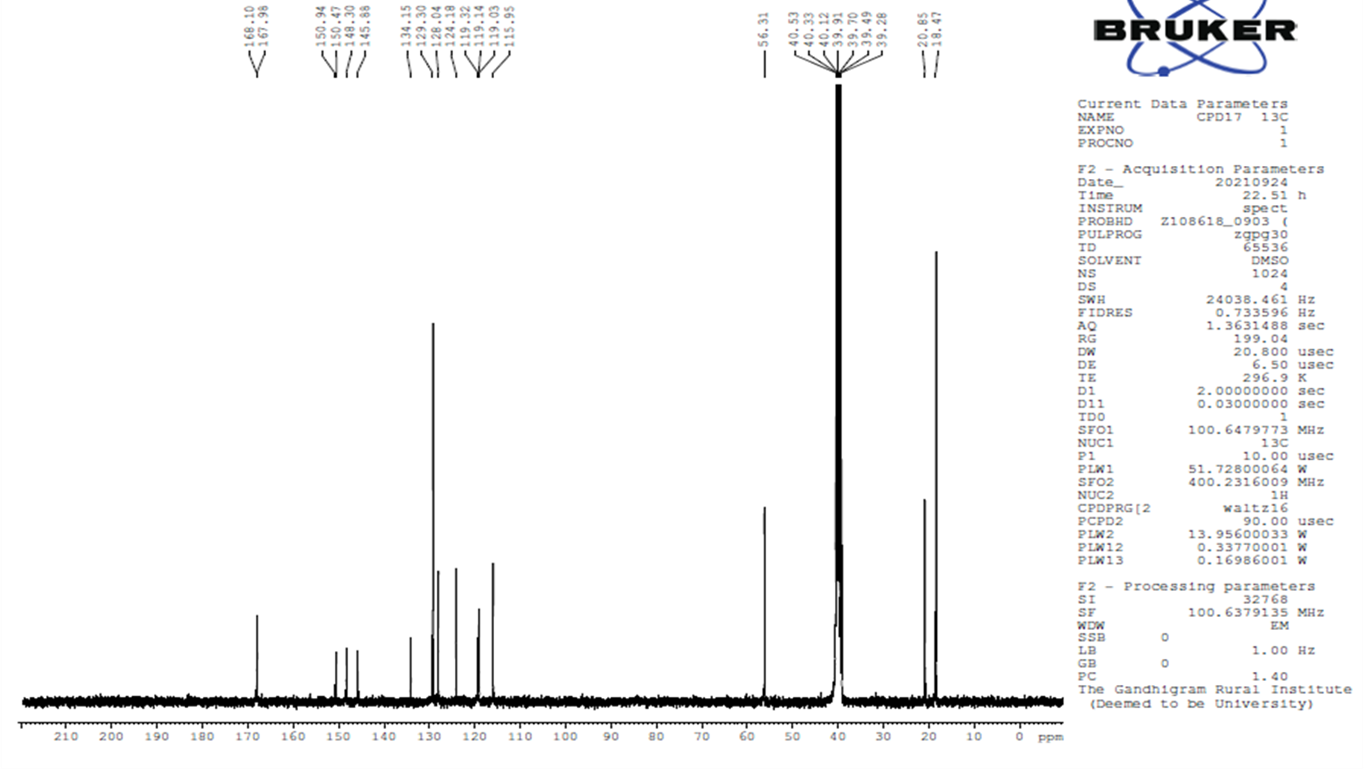


**Fig. S4:^13^C-NMR Spectrum of compound (I)**





**Fig. S5:Comparison of Experimental and Theoretical value of IR**
